# Activation‐Induced Killer Cell Immunoglobulin‐like Receptor 3DL2 Binding to HLA–B27 Licenses Pathogenic T Cell Differentiation in Spondyloarthritis

**DOI:** 10.1002/art.39515

**Published:** 2016-03-28

**Authors:** Anna Ridley, Hiroko Hatano, Isabel Wong‐Baeza, Jacqueline Shaw, Katherine K. Matthews, Hussein Al‐Mossawi, Kristin Ladell, David A. Price, Paul Bowness, Simon Kollnberger

**Affiliations:** ^1^ Botnar Research Centre, Nuffield Department of Orthopaedics, Rheumatology and Musculoskeletal Sciences, Oxford UniversityOxfordUK; ^2^ Isabel Wong‐Baeza, PhD: Botnar Research Centre, Nuffield Department of Orthopaedics, Rheumatology and Musculoskeletal Sciences, Oxford University, Oxford, UK, and National School of Biological Sciences, National Polytechnic InstituteMexico CityMexico; ^3^ QIMR, Berghofer Medical Research InstituteBrisbaneQueenslandAustralia; ^4^ Cardiff University School of MedicineCardiffUK; ^5^ Cardiff University School of Medicine, Cardiff, UK, and National Institute of Allergy and Infectious Diseases, NIHBethesdaMaryland; ^6^ Botnar Research Centre, Nuffield Department of Orthopaedics, Rheumatology, and Musculoskeletal Sciences, Oxford University, Oxford, UK, and Cardiff University School of MedicineCardiffUK

## Abstract

**Objective:**

** In the** **spondyloarthritides (SpA), increased numbers of CD4+ T cells express killer cell immunoglobulin‐like receptor 3DL2 (KIR‐3DL2). The aim of this study was to determine the factors that induce KIR‐3DL2 expression, and to characterize the relationship between HLA–B27 and the phenotype and function of KIR‐3DL2–expressing CD4+ T cells in SpA.**

**Methods:**

** In total, 34 B27+** **patients with SpA, 28 age‐ and sex‐matched healthy controls (20 B27− and 8 B27+), and 9 patients with rheumatoid arthritis were studied. KIR-3DL2 expression and other phenotypic characteristics of peripheral blood and synovial fluid CD4+ T cells were studied by flow cytometry, quantitative polymerase chain reaction, and Western blotting. T cell receptor clonality was determined by template‐switch anchored reverse transcription–polymerase chain reaction and sequencing analysis. Cytokines were measured by enzyme‐linked immunosorbent assay.**

**Results:**

Cellular activation induced KIR‐3DL2 expression on both naive and effector CD4+ T cells. KIR‐3DL2 binding to B27+ cells promoted expression of KIR‐3DL2, the Th17‐specific transcription factor retinoic acid receptor–related orphan nuclear receptor γt, and the antiapoptotic factor B cell lymphoma 2. KIR‐3DL2+CD4+ T cells in patients with ankylosing spondylitis were oligoclonal and enriched for markers of T cell activation and for the gut homing receptor CCR9. In the presence of B27+ antigen‐presenting cells, KIR‐3DL2+CD4+ T cells produced less interleukin‐2 (IL‐2) but more IL‐17. This effect was blocked by HC10, an antibody that inhibits the binding of KIR‐3DL2 to B27 heavy chains.

**Conclusion:**

KIR‐3DL2 binding to HLA–B27 licenses Th17 cell differentiation in SpA. These findings raise the therapeutic potential of targeting HLA–B27–KIR‐3DL2 interactions for the treatment of B27+ patients with SpA.

The spondyloarthritides (SpA) encompass a group of chronic inflammatory arthritic disorders typified by ankylosing spondylitis (AS), a condition in which up to 94% of affected individuals express the class I molecule HLA–B27 1. Genome‐wide association studies in AS have confirmed the key association with HLA–B27, and identified polymorphisms in genes associated with interleukin‐17 (IL‐17)–mediated immune responses, including IL‐1 receptor (IL‐1R) and IL‐23R 2, 3. Additional studies have shown raised levels of serum and synovial fluid IL‐17 in patients with SpA 4, 5, and raised numbers of circulating Th17 cells 6. However, the link between HLA–B27 and production of IL‐17 by immune cells is poorly understood.

We have previously shown that HLA–B27 exists as β_2_‐microglobulin (β_2_m)–free heavy chain forms, including heavy chain dimers 7, 8. B27 free heavy chain molecules bind more strongly than other HLA class I proteins to the killer cell immunoglobulin‐like receptor 3DL2 (KIR‐3DL2), promoting increased survival of natural killer (NK) cells and CD4+ T cells 9, 10, 11. Increased proportions of NK cells and CD4+ T cells expressing KIR‐3DL2 are present in individuals with SpA 10, 12, 13. These KIR‐3DL2+CD4+ T cells are enriched for the expression of IL‐17 and IL‐23R, as well as for other Th17 cell markers, and produce interferon‐γ (IFNγ). Moreover, the majority of all IL‐23R+ IL‐17–producing peripheral blood CD4+ T cells in patients with SpA reside in the minority KIR‐3DL2+ T cell compartment 13.

In the present study, we show that CD4+ T cells up‐regulate cell surface KIR‐3DL2 expression upon activation. Subsequent binding of KIR‐3DL2 to HLA–B27 maintains the expression of KIR‐3DL2 and promotes T cell survival and Th17 cell differentiation. In addition, we provide evidence that this mechanism is proinflammatory in SpA.

## PATIENTS AND METHODS

### Patient and control samples

Samples of heparinized venous blood (25 ml) were obtained from 34 B27+ patients with SpA, 33 with definite AS fulfilling the modified New York criteria 14, 20 B27− and 8 B27+ healthy controls, and 9 disease controls with rheumatoid arthritis (RA). Patient and control demographics and medications are shown in Supplementary Table 1 (available on the *Arthritis & Rheumatology* web site at http://onlinelibrary.wiley.com/doi/10.1002/art.39515/abstract). Ethics permission was obtained from the Central Office for Research Ethics Committees (approval number 06/Q1606/139), and all subjects gave their individual written informed consent to participate.

### Separation of CD4+ T cells

Peripheral blood and synovial fluid mononuclear cells were isolated by density‐gradient centrifugation. Total or naive (CD45RO−) CD4+ T cells were separated by negative selection on magnetic beads (Miltenyi Biotec). CD4+ T cells were activated either with anti‐CD2/CD3/CD28 beads (Miltenyi Biotec) or with 125 ng/ml phorbol myristate acetate (PMA) and 1 μg/ml ionomycin (Sigma).

### CD4+ T cell coculture with antigen‐presenting cells (APCs)

LBL.721.221 and LBL.721.220 APC lines transfected with HLA–B*27:05 and other class I molecules were used, as has been described previously 8, 15. Irradiated LBL.721 APCs (100,000 cells) were incubated with 200,000 naive or total CD4+ T cells (labeled with 5,6‐carboxyfluorescein succinimidyl ester [Life Technologies]), followed by incubation with 100 ng/ml staphylococcal enterotoxin B (SEB; Sigma), as previously described 13. After 5–8 days, the cells were analyzed by flow cytometry, and supernatants were collected for enzyme‐linked immunosorbent assays (ELISAs; eBioscience) to detect IL‐2 and IL‐17A. Irradiated APCs were removed after coculture, using a Dead Cell Removal kit (Miltenyi Biotec), and enriched T cells were processed for RNA extraction and quantitative polymerase chain reaction (qPCR).

For Th17 cell differentiation experiments, naive T cells were cultured for 8 days at a 1:5 ratio with anti‐CD2/CD3/CD28 beads or at a 1:2 ratio with transfected LBL.721.220 cells and 10 ng/ml SEB along with 20 IU/ml recombinant human IL‐2 (rhIL‐2; PeproTech) and 10 ng/ml rhIL‐1, IL‐6, and IL‐23 (PeproTech) for 8 days. Supernatants were harvested for cytokine assay by ELISA, and T cells were analyzed by fluorescence‐activated cell sorting (FACS) intracellular cytokine staining for the production of IL‐2, IFNγ, and IL‐17. Cells were treated with monensin (BD Biosciences) overnight before being stained for IL‐2 and IFNγ, using protocols adapted from previously described methods 13. Cells were stimulated for 6 hours with monensin and PMA and ionomycin, and then stained for IL‐17. For antibody‐blocking experiments, cells were incubated with isotype control antibodies (IgG1/IgG2a; BioLegend) or HC10 (anti–class I heavy chain), HD6 (anti–B27 heavy chain), ME1 (anti–HLA–B27; binds β_2_m‐associated B27), or DX31 (anti–KIR‐3DL2) antibodies at 10 μg/ml. Antibodies were replenished after 3 days of cell culture.

### T cell receptor (TCR) sequencing

Clonotypic analysis of isolated CD4+ T cell populations was performed as described previously, with minor modifications 16. Viable KIR‐3DL2+ or KIR‐3DL2− CD3+CD4+ T cells were sorted ex vivo into 1.5‐ml microtubes (10,000 cells per tube; Sarstedt) containing 100 μl RNAlater (Applied Biosystems) using a FACSAria II flow cytometer (BD Biosciences). Unbiased amplification of all expressed *TRB* gene products was conducted using a template‐switch anchored reverse transcription–PCR with a 3 ′ constant region primer (5 ′‐TGGCTCAAACAAGGAGACCT‐3 ′). Amplicons were subcloned, sequenced, and analyzed as described previously 17. The ImMunoGeneTics nomenclature 18 was used for all sequenced gene products.

### Flow cytometry

The following antibodies were used for flow cytometric analyses: IgG2a and anti‐DX31 (AF647 in‐house conjugates; provided by Jo Philips from DNAX Research Institute); PerCP‐conjugated anti‐CD3, fluorescein isothiocyanate (FITC)–conjugated anti‐CD45RO, FITC‐conjugated anti‐CD69, FITC‐conjugated anti‐OX40, and phycoerythrin (PE)–conjugated anti‐CCR6 (BD Biosciences); PE‐conjugated anti–IL‐17A and PE–Cy7–conjugated anti‐CD4 (eBioscience); and FITC‐conjugated anti–IL‐1R, FITC‐conjugated anti‐CCR9, and FITC‐conjugated anti–IL‐23R (R&D Systems). Dead cells were excluded using an allophycocyanin–Cy7–conjugated Live/Dead stain (Invitrogen). Intracellular cytokine staining was performed using PE‐conjugated anti–IL‐2 (eBioscience), using protocols adapted from previously described methods 13. At least 200,000 cells/sample were analyzed by flow cytometry, using an LSR Fortessa (BD Biosciences). Data were analyzed using FlowJo software (version 8.6.6; TreeStar).

### Gene expression analysis by qPCR and Western blotting

RNA extraction was performed with an Absolutely RNA Microprep kit (Agilent Technologies), and complementary DNA was synthesized with a SuperScript VILO kit (Invitrogen). Gene expression levels of *Bcl2*, *CCR6*, *CD3d*, *IL1R*, *IL23R*, *RORC* (for retinoic acid receptor–related orphan nuclear receptor γt [RORγt]), and *TBX21* (for T‐bet) were evaluated with TaqMan Gene Expression assays (Applied Biosystems). Amplification reactions were performed in a 7500 Fast Real‐Time PCR system (Applied Biosystems), comprising 1 cycle at 95°C for 20 seconds, followed by 40 cycles at 95°C for 3 seconds and 60°C for 30 seconds. The ΔΔC_t_ method was used to determine the gene expression values relative to those for CD3d. Sodium dodecyl sulfate–polyacrylamide gel electrophoresis and Western blotting were performed as described previously 8. Western blots were probed with rabbit polyclonal anti–KIR‐3DL2 antibodies (Pierce) or mouse anti–β‐actin monoclonal antibodies (mAb) (Sigma) and horseradish peroxidase–conjugated goat anti‐rabbit or anti‐mouse immunoglobulins (Dako).

### Statistical analysis

Statistical analysis was conducted using GraphPad Prism software version 4. *P* values were calculated using Student's *t*‐test or one‐way analysis of variance with Bonferroni correction, as indicated.

## RESULTS

### Induction of KIR‐3DL2 expression upon CD4+ T cell activation, and correlation between KIR‐3DL2 expression and T cell activation in patients with SpA

**Figure 1:**
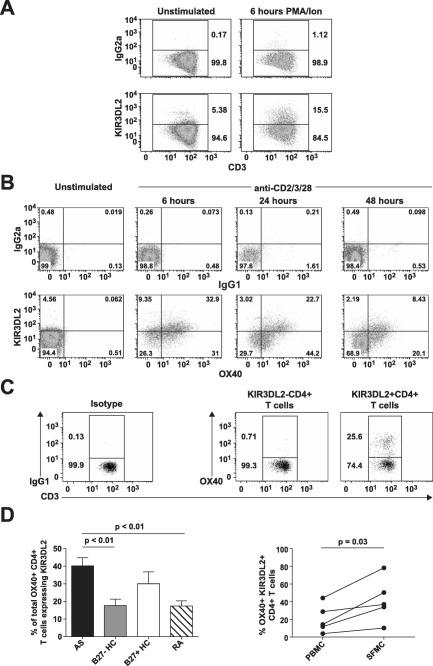
Expression of killer cell immunoglobulin‐like receptor 3DL2 (KIR‐3DL2) is induced upon CD4+ T cell activation, and KIR‐3DL2 expression is correlated with T cell activation in patients with spondyloarthritis (SpA). **A** and **B,** Flow cytometry plots show staining of purified CD4+ T cells from a healthy control (HC) for the expression of KIR‐3DL2 before and 6 hours after stimulation with phorbol myristate acetate (PMA) and ionomycin (Ion) **(A)** or the expression of KIR‐3DL2 and OX40 before and 6, 24, or 48 hours after stimulation with anti‐CD2/CD3/CD28 beads **(B)**. Representative results from 1 of 3 independent experiments are shown. **C,** Flow cytometry plots show staining for the expression of OX40 on peripheral blood KIR‐3DL2− or KIR‐3DL2+ CD4+ T cells from a patient with ankylosing spondylitis (AS). IgG1 and IgG2a antibodies were used as isotype controls in the flow cytometry analyses. **D,** The percentage of OX40+CD4+ T cells expressing KIR‐3DL2 was determined in the peripheral blood from patients with AS (n = 15), B27− healthy controls (n = 15), B27+ healthy controls (n = 8), and patients with rheumatoid arthritis (RA) (n = 9) (left) or in matched peripheral blood mononuclear cell (PBMC) and synovial fluid mononuclear cell (SFMC) samples from 5 patients with SpA (right). Values are the mean ± SD. *P* values were determined by analysis of variance.

We previously detected expansion of Th17 cells expressing KIR‐3DL2 in patients with SpA 13. In this study, we sought to determine whether T cell activation was sufficient to induce KIR‐3DL2 expression. After stimulation of human CD4+ T cells with PMA and ionomycin for 6 hours, KIR‐3DL2 expression was markedly increased (Figure 1A). KIR‐3DL2 expression on CD4+ T cells was also increased after stimulation with anti‐CD2/CD3/CD28 beads (Figure 1B) and in the presence of superantigen (results not shown). The increase in KIR‐3DL2 expression was associated with coexpression of the T cell activation marker OX40 (Figure 1B).

We next determined whether KIR‐3DL2 expression was correlated with T cell activation in patients with SpA. In B27+ patients with SpA, KIR‐3DL2+CD4+ T cells were highly enriched for OX40 expression, a marker of CD4+ T cells that have recently experienced antigen (Figure 1C). The proportion of OX40+CD4+ T cells expressing KIR‐3DL2 was significantly greater in patients with SpA compared to either B27− healthy control subjects or RA disease controls (Figure 1D), but was not significantly different between patients with SpA and B27+ healthy controls. The mean ± SEM proportions of total live CD4+ T cells expressing OX40 were 1.5 ± 0.5%, 2.8 ± 1.1%, 1.6 ± 0.3%, and 2.7 ± 0.8% in patients with SpA, B27+ healthy controls, B27− healthy controls, and patients with RA, respectively. Thus, the increased proportions of KIR‐3DL2+ OX40+CD4+ T cells observed in patients with SpA and B27+ healthy controls occurred as a consequence of the increased percentages of KIR‐3DL2+CD4+ T cells in these groups.

We also examined KIR‐3DL2 expression on CD4+ T cells from synovial fluid. KIR‐3DL2+CD4+ T cells were further enriched for OX40 expression in matched synovial fluid samples from the affected joints of 5 patients with SpA (Figure 1D).

### Up‐regulation of KIR‐3DL2 expression on naive CD4+ T cells following activation

**Figure 2:**
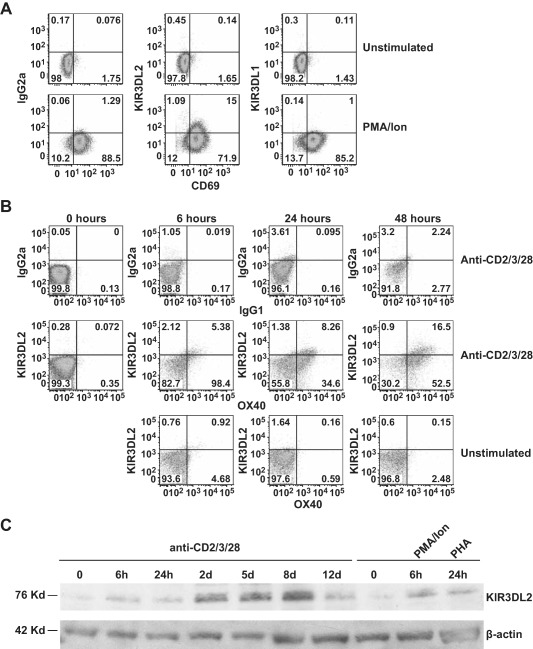
Expression of KIR‐3DL2 is induced by activation of naive CD4+ T cells. **A,** Flow cytometry plots show staining for KIR‐3DL2 or KIR‐3DL1 and CD69 in purified naive CD4+ T cells from a patient with AS before and 6 hours after stimulation with PMA and ionomycin. **B,** Flow cytometry plots show staining for KIR‐3DL2 and OX40 in naive CD4+ T cells from a healthy control before and 6, 24, or 48 hours after stimulation with anti‐CD2/CD3/CD28 beads as compared to unstimulated control cells. IgG1 and IgG2a antibodies were used as isotype controls in the flow cytometry analyses. **C,** Western blots show the expression of KIR‐3DL2, as compared to β‐actin, before or after activation of purified naive CD4+ T cells from a healthy control with anti‐CD2/CD3/CD28 beads for 6 hours, 24 hours, 2 days, 5 days, 8 days, or 12 days, with PMA and ionomycin for 6 hours, or with phytohemagglutinin (PHA) for 24 hours. Representative results from 1 of 3 independent experiments are shown. See Figure 1 for other definitions.

We next isolated naive CD4+ T cells (>96% purity) from healthy donors and patients with AS. Fewer than 0.5% of naive CD4+ T cells from patients with AS expressed KIR‐3DL2 (see Supplementary Figure 1, http://onlinelibrary.wiley.com/doi/10.1002/art.39515/abstract). Activation with either PMA and ionomycin or anti‐CD2/CD3/CD28 beads induced expression of KIR‐3DL2, but not KIR‐3DL1, on naive T cells (Figure 2A and results not shown). Up‐regulated expression of KIR‐3DL2 was induced more slowly with anti‐CD2/CD3/CD28 beads than with PMA and ionomycin, reaching a maximum level of expression at 48 hours (Figure 2B). Activated naive KIR‐3DL2+CD4+ T cells also expressed CD69 and OX40 (Figures 2A and B). Similar results were obtained using naive CD4+ T cells from cord blood (results not shown), confirming that KIR‐3DL2 expression could also be induced on antigen‐inexperienced naive T cells.

Western blot analysis showed that freshly isolated naive T cells expressed low levels of KIR‐3DL2 (Figure 2C). Increased KIR‐3DL2 protein expression after activation with any of the stimuli was detected by Western blotting from as early as 6 hours, reaching a maximum level of expression with anti‐CD2/CD3/CD28 bead stimulation at 8 days.

### Oligoclonal TCR repertoire of KIR‐3DL2+CD4+ T cells in patients with SpA

**Figure 3:**
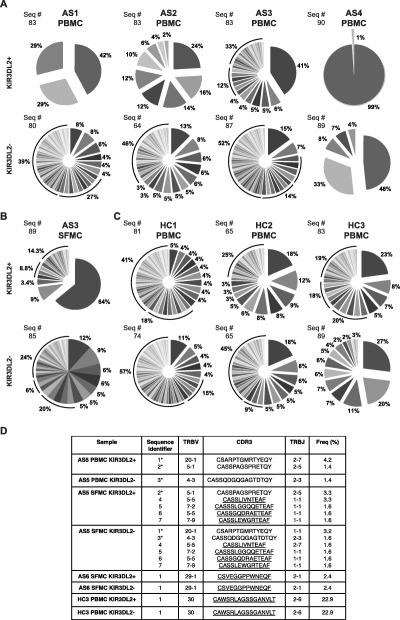
KIR‐3DL2+CD4+ T cells from patients with AS are oligoclonal. **A**–**C,** Pie charts show the distribution of distinct T cell receptor (TCR) sequences of the KIR‐3DL2+ and KIR‐3DL2− CD4+ T cell subsets of PBMCs from 4 patients with AS **(A),** SFMCs from 1 patient with AS **(B)**, or PBMCs from 3 B27− healthy controls **(C)**. The numbers of sequences (Seq #) are also shown. **D,** TCR sequences of the KIR‐3DL2+ and KIR‐3DL2− T cell subsets were identical in matched PBMCs and SFMCs from 1 patient with AS (patient AS5), SFMCs from 1 patient with AS (patient AS6), and PBMCs from a B27− healthy control (HC3). Sequences marked with an **asterisk** were found in matched PBMC and SFMC samples. Identical sequences are underlined. CDR3 = third complementarity‐determining region; Freq = frequency (see Figure 1 for other definitions).

We next compared the TCR sequences from equal numbers of KIR‐3DL2+ and KIR‐3DL2− CD4+ T cells that had been isolated directly ex vivo from the peripheral blood of patients with SpA and healthy controls (>96% purity, as determined by flow cytometry [see Supplementary Figure 2, http://onlinelibrary.wiley.com/doi/10.1002/art.39515/abstract]). All of the TCR sequences identified in CD4+ T cells from patients with SpA and healthy controls are listed in Supplementary Table 2 (http://onlinelibrary.wiley.com/doi/10.1002/art.39515/abstract). KIR‐3DL2+CD4+ T cells from B27+ patients with SpA (n = 4) displayed an oligoclonal TCR repertoire, with evidence of skewing toward dominant clonotypes (Figure 3A). Synovial fluid KIR‐3DL2+CD4+ T cells from a patient with AS were also oligoclonal (Figure 3B). In contrast, KIR‐3DL2+CD4+ T cells purified from healthy controls exhibited more diverse TCR repertoires (Figure 3C).

Overall, the mean ± SEM numbers of unique TCR sequences in the KIR‐3DL2+ and KIR‐3DL2− CD4+ T cell populations in patients with SpA (n = 5) were 19.8 ± 10.5 and 39.2 ± 9.43, respectively (*P* = 0.04, by paired *t*‐test). In contrast, the mean ± SEM numbers of unique TCR sequences in the KIR‐3DL2+ and KIR‐3DL2− CD4+ T cell populations in the control group (n = 3) were not significantly different (36.67 ± 7.06 and 33 ± 11.02, respectively). Identical clonotypes were detected across the KIR‐3DL2+ and KIR‐3DL2− T cell compartments in 3 individuals (Figure 3D and Supplementary Table 2, http://onlinelibrary.wiley.com/doi/10.1002/art.39515/abstract). Given their identical nucleic acid sequences, it is likely that these T cells are the progeny of individual sister clones that dynamically regulate KIR‐3DL2 expression.

### Promotion of KIR‐3DL2 expression and Th17 cell differentiation by CD4+ T cell activation in the presence of HLA–B27

We next activated naive CD4+ T cells from patients with AS and healthy controls with SEB in the presence of LBL.721.221 or LBL.721.220 B cell lines, each of which expresses similar surface densities of different HLA class I molecules. Both LBL.721.220 B27+ cells and LBL.721.221 B27+ cells express high levels of B27 dimers and free heavy chains 7, 8. Moreover, the KIR‐3DL2 receptor expressed by NK cells or reporter cells binds equally strongly to B27 dimers and free heavy chains expressed by transfected LBL.721.220 and LBL.721.221 cells 9.

**Figure 4:**
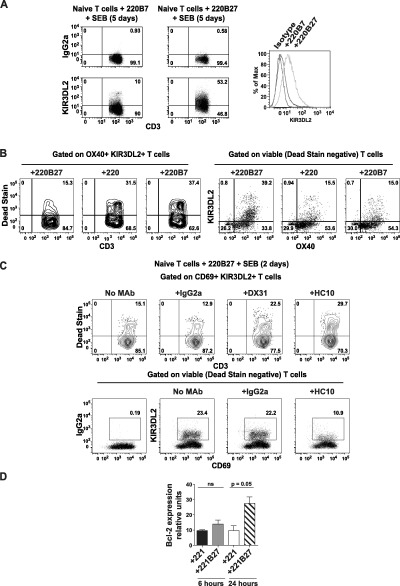
Binding of KIR‐3DL2 to B27 heavy chains promotes the survival of activated naive CD4+ T cells. **A,** Left, Flow cytometry plots show staining for KIR‐3DL2 or IgG2a isotype control in CD45RO+ T cells from a patient with AS after 5 days of stimulation with LBL.721.220 cells (transfected with HLA–B7 [220B7] or HLA–B27 [220B27]) and staphylococcal enterotoxin B (SEB). Right, KIR‐3DL2 expression in cells under each condition is shown as the percentage of maximum (Max). **B,** OX40+KIR‐3DL2+ naive CD4+ T cells from a healthy control were analyzed by Live/Dead staining to exclude dead cells (left) and identify viable cells (right), after 5 days of stimulation with the indicated LBL.721.220 cells and SEB. Nontransfected LBL.721.220 cells (220) were used as a transfection control. Isotype control monoclonal antibody (mAb) did not stain (results not shown). **C,** CD69+KIR‐3DL2+ T cells among naive CD4+ T cells from a healthy control were analyzed by Live/Dead staining to exclude dead cells (top) and identify viable cells (bottom), after 2 days of stimulation with LBL.721.220 B27+ cells and SEB with or without the indicated mAb. In the flow cytometry analyses, representative results from 1 of 3 independent experiments are shown. **D,** Bcl‐2 expression was assessed by quantitative polymerase chain reaction in healthy control naive CD4+ T cells cocultured with the indicated LBL.721.221 cells and SEB for 6 or 24 hours. Results are the mean ± SEM of 5 independent experiments. *P* values were determined by analysis of variance. NS = not significant (see Figure 1 for other definitions).

Although initial induction of KIR‐3DL2 on CD4+ T cells occurred independently of the expressed HLA class I allele (results not shown), higher levels of KIR‐3DL2 expression were maintained over time in the presence of B27+ APCs (Figure 4A). Naive T cells activated with LBL.721.220 B27+ cell transfectants also expressed higher levels of KIR‐3DL2 messenger RNA (mRNA), as assayed by qPCR (see Supplementary Figure 3, http://onlinelibrary.wiley.com/doi/10.1002/art.39515/abstract). In all cases, activated naive KIR‐3DL2+ cells expressed the effector memory T cell marker CD45RO (results not shown). Similar results were obtained with naive CD4+ T cells from healthy controls (see Supplementary Figure 4, http://onlinelibrary.wiley.com/doi/10.1002/art.39515/abstract) and unsorted (bulk) CD4+ T cells (results not shown). Furthermore, these cells survived better after activation in the presence of LBL.721.220 B27+ cells, compared to stimulation with parental or control HLA class I–transfected LBL.721.220 cells (Figure 4B).

The class I heavy chain antibody HC10 inhibited the expression of KIR‐3DL2 and concomitantly decreased the numbers of viable naive or bulk CD4+ T cells stimulated with LBL.721.220 B27+ cells (Figure 4C and results not shown). The KIR‐3DL2–specific antibody DX31 also inhibited survival of activated naive CD4+ T cells (Figure 4C and Supplementary Figure 4, http://onlinelibrary.wiley.com/doi/10.1002/art.39515/abstract). In contrast, the ME1 antibody, which recognizes β_2_m‐associated HLA–B27, did not affect KIR‐3DL2 expression and cell survival of naive T cells activated with LBL.721.220 B27+ cells (Supplementary Figure 4, http://onlinelibrary.wiley.com/doi/10.1002/art.39515/abstract). In addition, activation of naive CD4+ T cells with LBL.721.221 B27+ cells significantly up‐regulated *Bcl2* transcript expression at 24 hours (Figure 4D). Thus, activation with B27+ APCs promotes naive CD4+ T cell survival and expression of KIR‐3DL2.

### Inhibition of CD4+ T cell IL‐2 production by KIR‐3DL2 interactions with HLA–B27 leading to Th17 cell differentiation

**Figure 5:**
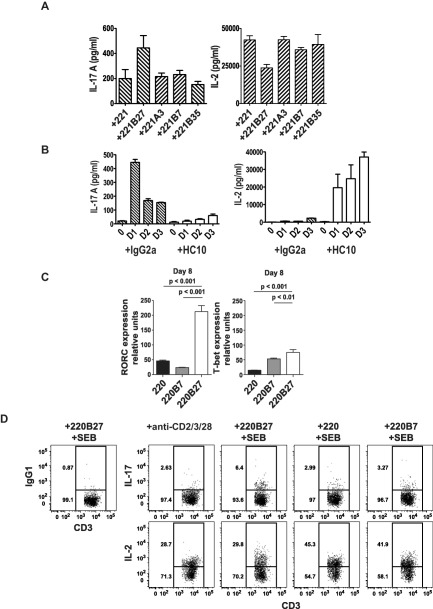
Interactions of KIR‐3DL2 with B27+ antigen‐presenting cells inhibit the production of interleukin‐2 (IL‐2) and promote Th17 cell differentiation. **A,** Secretion of IL‐17A and IL‐2 was assessed in CD4+ T cells from healthy controls (n = 6) cocultured for 5 days with LBL.721.221 cells (left nontransfected [221] or transfected with the indicated HLA class I molecules) and SEB. Values for T cells stimulated with LBL.721.221 B27+ cells were significantly different (*P* < 0.05 by analysis of variance) from those in T cells cocultured under the other conditions. **B,** Production of IL‐17A and IL‐2 was assessed in CD4+ T cells from healthy controls (n = 3) cocultured for up to 3 days with LBL.721.220 B27+ cells, SEB, and HC10 antibodies or IgG2a as isotype control. **C,** Expression of the retinoic acid receptor–related orphan nuclear receptor γt gene *RORC* and the T‐bet gene *TBX21* was assessed in naive CD4+ T cells isolated from the peripheral blood of a healthy control after coculture with the indicated LBL.721.220 cells and SEB for 8 days. Gene expression was normalized to that of CD3. Representative results from 4 independent experiments are shown. In **A–C,** values are the mean ± SD. **D,** Representative flow cytometry plots show the proportions of viable IL‐17+ and IL‐2+ naive T cells after 5 days of stimulation with Th17 cytokines (IL‐1, IL‐6, and IL‐23) along with anti‐CD2/CD3/CD28 beads or with the indicated LBL.721.220 cells and SEB. IgG1 was used as the isotype control. See Figure 1 for other definitions.

We next examined the functional effect of KIR‐3DL2 binding to HLA–B27 on CD4+ T cell lineage differentiation. It has been established that IL‐2 inhibits the production of IL‐17, and we previously demonstrated that KIR‐3DL2 binding to HLA–B27 on LBL.721.220 B27+ cells promotes IL‐17 production 13. Moreover, KIR ligation by HLA class I inhibits IL‐2 production (19). Thus, we reasoned that KIR‐3DL2 binding to B27 dimers on transfected cells could promote an IL‐17–secreting phenotype by reducing CD4+ T cell production of IL‐2. Consistent with this notion, LBL.721.221 B27+ cells consistently stimulated greater production of IL‐17 and lower production of IL‐2 by activated T cells than did parental LBL.721.221 cells or control transfectants (Figure 5A).

The increased production of IL‐17 observed following stimulation of CD4+ T cells with LBL.721.221 B27+ and LBL.721.220 B27+ cells was inhibited by HLA class I heavy chain (HC10) antibodies and anti–KIR‐3DL2 (DX31) antibodies (see ref. 13 and Supplementary Figure 5, http://onlinelibrary.wiley.com/doi/10.1002/art.39515/abstract). HC10 antibodies inhibited IL‐17 production and promoted IL‐2 production by CD4+ T cells that had been stimulated with LBL.721.220 B27+ cells (Figure 5B). We consistently observed lower production of IL‐2 by CD4+ T cells when the cells were stimulated with LBL.721.220 B27+ cells as compared to when they were stimulated with LBL.721.221 B27+ cells. Compared to LBL.721.221 B27+ cells, LBL.721.220 B27+ cells express higher levels of B27 free heavy chains, which are capable of binding to KIR‐3DL2 8.

To extend this observation, we studied the effect of superantigen activation on CD4+ T cell differentiation. Activation of naive T cells in the presence of B27+ APCs consistently increased *RORC* gene expression (a key Th17‐specific transcription factor) by naive CD4+ T cells, as compared to that following stimulation with parental LBL.721.220 cells or control transfectants (Figure 5C). The effect on *RORC* expression was apparent from 5 days after activation (results not shown).

We further reasoned that increased expression of *RORC* could promote the differentiation of Th17 cells. Accordingly, we compared IL‐17 production by naive T cells stimulated with LBL.721.220 B27+ cells and LBL.721.220 control transfectants, or anti‐CD2/CD3/CD28 beads in the presence of the Th17 cytokines IL‐23, IL‐1, and IL‐6, together with low concentrations of IL‐2. Activation with LBL.721.220 B27+ cells and Th17 cytokines promoted greater production of IL‐17 by naive T cells compared to that with the other stimuli (Figure 5D and Supplementary Figures 5 and 6, http://onlinelibrary.wiley.com/doi/10.1002/art.39515/abstract). Naive T cells activated with LBL.721.220 B27+ cells produced less IL‐2 compared to that after stimulation with control LBL.721.220 cells (Figure 5D). IL‐17 production by naive T cells activated with LBL.721.220 B27+ cells and Th17 cytokines was inhibited by HC10 and DX31 antibodies (Supplementary Figures 5 and 6, http://onlinelibrary.wiley.com/doi/10.1002/art.39515/abstract). FACS staining showed that naive CD4+ T cells activated with LBL.721.220 B27+ cells also produced less IL‐2 compared to that produced by control LBL.721.220‐stimulated naive CD4+ T cells (Figure 5D).

We also studied the effect of physiologic levels of HLA–B27 expressed by Epstein‐Barr virus–transformed B cell lines on the differentiation of naive T cells activated with Th17 cytokines and superantigen. Differentiation of naive T cells with B27‐expressing B cell lines promoted greater production of IL‐17 than differentiation with cell lines that did not express B27 (Supplementary Figure 7, http://onlinelibrary.wiley.com/doi/10.1002/art.39515/abstract). Production of IL‐17 by naive T cells stimulated with B27+ B cell lines was inhibited by both anti–class I heavy chain and anti–KIR‐3DL2 mAb (Supplementary Figure 7, http://onlinelibrary.wiley.com/doi/10.1002/art.39515/abstract).

We have previously shown that the mAb HD6, which recognizes B27 heavy chain dimers, inhibits IL‐17 production by CD4+ T cells from patients with SpA 20. Compared to the HC10 mAb, HD6 has a lower avidity for B27 free heavy chains and recognizes a distinct epitope (Marroquin O, et al: unpublished observations). Our previous results have shown that HD6 inhibits the survival of KIR‐3DL2+ T cells 9. In this study, we found that HD6 inhibited the survival of KIR‐3DL2+CD4+ T cells in the peripheral blood of patients with SpA (see Supplementary Figure 8, http://onlinelibrary.wiley.com/doi/10.1002/art.39515/abstract).

Collectively, these results show that the interaction between KIR‐3DL2 and B27+ APCs can skew the differentiation of CD4+ T cells toward a Th17 cell phenotype.

### Enrichment for gut homing and Th17 cell differentiation markers of KIR‐3DL2+CD4+ T cells from patients with SpA

**Figure 6:**
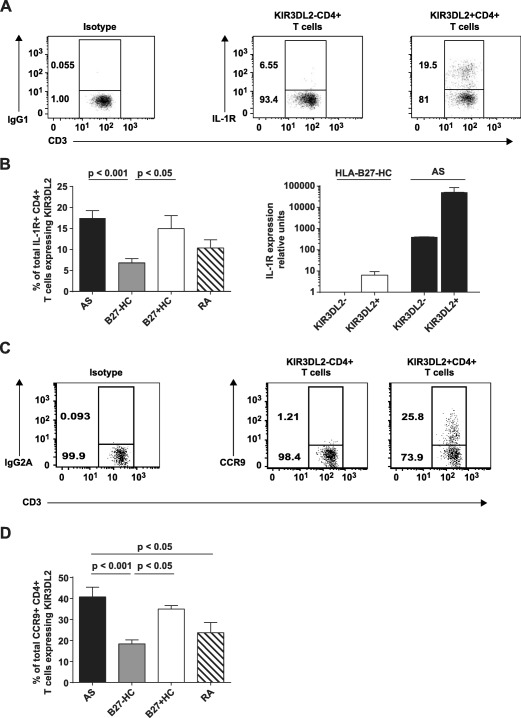
KIR‐3DL2+CD4+ T cells from B27+ patients with AS are enriched for the expression of interleukin‐1 receptor (IL‐1R) and CCR9. **A,** Flow cytometry plots show IL‐1R expression by peripheral blood KIR‐3DL2− and KIR‐3DL2+ CD4+ T cells from a representative patient with AS. IgG1 was used for isotype control staining. **B,** Left, Percentages of IL‐1R+CD4+ T cells expressing KIR‐3DL2 were determined in the peripheral blood of patients with AS (n = 15), B27− healthy controls (n = 15), B27+ healthy controls (n = 8), and patients with RA (n = 8). Right, Gene expression of *IL1R* was assessed by quantitative polymerase chain reaction in KIR‐3DL2+ and KIR‐3DL2− CD4+ peripheral blood T cells from a B27− healthy control and a patient with AS. Gene expression was normalized to that of CD3. Representative results from 3 independent experiments are shown. **C,** Flow cytometry plots show CCR9 expression by peripheral blood KIR‐3DL2− and KIR‐3DL2+ CD4+ T cells from a representative patient with AS. IgG2a was used for isotype control staining. **D,** Percentages of CCR9+CD4+ T cells expressing KIR‐3DL2 were determined in the peripheral blood of B27+ patients with AS (n = 15), B27− healthy controls (n = 15), B27+ healthy controls (n = 8), and patients with RA (n = 8). Values in **B** and **D** are the mean ± SEM. *P* values were determined by analysis of variance. See Figure 1 for other definitions.

We previously found that KIR‐3DL2+CD4+ T cells in patients with SpA are enriched for the expression of the Th17 cell markers IL‐23R and CCR6 13. To extend these findings, we studied additional differentiation and homing markers by flow cytometry and qPCR. Peripheral blood KIR‐3DL2+ CD4+ T cells from patients with SpA were enriched for both IL‐23R and CCR6 expression, at both the protein and transcript levels, as compared to KIR‐3DL2− CD4+ T cells both from patients with SpA and from B27− healthy controls (see Supplementary Figure 9, http://onlinelibrary.wiley.com/doi/10.1002/art.39515/abstract). Representative flow cytometry data from a patient with AS revealed an enrichment of IL‐1R expression on peripheral blood KIR‐3DL2+CD4+ T cells (Figure 6A). Significantly greater expression of KIR‐3DL2 on IL‐1R+CD4+ T cells was detected in B27+ patients with AS and B27+ healthy controls compared to B27− healthy controls and RA disease controls (Figure 6B). KIR‐3DL2+CD4+ T cells from patients with SpA also expressed higher levels of IL‐1R mRNA (Figure 6B). Notably, we did not observe significant differences in cell surface expression levels of the Th17 phenotypic markers on CD4+ T cells lacking KIR‐3DL2 in any of the groups studied.

Increased expression of the gut chemokine receptor CCR9 was also detected on peripheral KIR‐3DL2+CD4+ T cells from patients with AS and B27+ healthy controls (Figures 6C and D). Remarkably, 40% of all circulating CCR9+CD4+ T cells in patients with SpA expressed KIR‐3DL2 (Figure 6D). Moreover, CCR9 expression on synovial fluid KIR‐3DL2+CD4+ T cells was elevated in patients with SpA (results not shown). Similar to our observations with Th17 phenotypic markers, we did not observe significant differences in the level of CCR9 expression on CD4+ T cells lacking KIR‐3DL2 expression in any of the groups studied. Thus, the increased proportions of KIR‐3DL2+IL‐1R+ and CCR9+ CD4+ T cells observed in patients with SpA occurred as a consequence of the increased percentages of KIR‐3DL2+CD4+ T cells.

KIR‐3DL2+CD4+ T cells also were found to produce more IL‐6 (Supplementary Figure 9, http://onlinelibrary.wiley.com/doi/10.1002/art.39515/abstract), but not IL‐5 or IL‐10 (results not shown), in response to stimulation with anti‐CD2/CD3/CD28 beads.

## DISCUSSION

In this study, we showed that KIR‐3DL2 is transiently up‐regulated on the surface of CD4+ T cells upon activation. Subsequent binding of KIR‐3DL2 to B27 free heavy chains on the surface of APCs maintains receptor expression and promotes Th17 cell differentiation and IL‐17 production. These results suggest a mechanism whereby HLA–B27 predisposes individuals to the development of SpA. Moreover, the presence of oligoclonal KIR‐3DL2+CD4+ T cell populations expressing markers of activation and Th17 cell lineage commitment in the blood and joints of patients with SpA provides strong evidence that this process occurs in vivo.

KIR expression on CD8+ T cells following TCR ligation has been reported previously 21. Similarly, the results of this study show, for the first time, that KIR‐3DL2 is expressed by CD4+ T cells in response to activation. Freshly isolated naive CD4+ T cells expressed low levels of KIR‐3DL2 both transcriptionally and in protein form. However, surface up‐regulation of KIR‐3DL2 was detected within 6 hours of activation, indicating that rapid translational activity is induced upon stimulation. It is notable in this regard that the presence of KIR transcripts has been detected previously in T cells lacking surface expression of the encoded receptor 22. The KIR‐3DL2 promoter region is unique among the KIR family gene promoters, as it incorporates multiple potential binding sites for NF‐κB, which could play a role in activation‐induced expression 23.

The presence of B27+ APCs both maintained KIR‐3DL2 expression by CD4+ T cells and skewed differentiation toward a Th17 or Th1/Th17 phenotype. We consider this a form of “licensing” in terms of lineage commitment. T cells with both a Th1 and a Th17 phenotype have been observed in patients with Crohn's disease 24 and in those with juvenile idiopathic arthritis 25. How might the interaction between KIR‐3DL2 and HLA–B27 drive CD4+ T cells to produce IL‐17? We propose that KIR‐3DL2 binding to noncanonical B27 free heavy chains enhances RORγt (and Bcl‐2) expression, perhaps through a net decrease in CD3‐transduced signaling strength. Indeed, weak TCR signals promote Th17 responses 26. Consistent with this possibility, we observed reduced production of IL‐2 by CD4+ T cells stimulated with B27+ APCs, a phenomenon that correlated temporally with the induction of KIR‐3DL2. It is also established that IL‐2 inhibits the production of IL‐17 by CD4+ T cells 27. Accordingly, inhibition of KIR‐3DL2 binding to B27 free heavy chains increased T cell production of IL‐2 and decreased production of IL‐17 by CD4+ T cells. Several observations further support this proposed mechanism. First, the ZAP70 kinase mutation in SKG mice attenuates TCR signaling and predisposes to a disease resembling SpA, through increased production of IL‐17 and decreased production of IL‐2 28. Second, reduced production of IL‐2 by T cells has been observed in AS 29, 30. Third, dendritic cells from HLA–B27–transgenic rats display impaired conjugate formation and promote Th17 responses 31, 32. These findings are consistent with an immunoregulatory role for cell surface B27 free heavy chains, which are expressed by transgenic rat dendritic cells 33.

We also show that KIR‐3DL2+CD4+ T cells are enriched for expression of OX40 and for IL‐6 production. Both IL‐6 and OX40–OX40L interactions positively regulate Th17 responses 34. Notably, IL‐6 levels are correlated with the levels of inflammation markers in SpA 35.

The oligoclonal and highly skewed TCR repertoires observed in KIR‐3DL2+CD4+ T cell populations from patients with SpA further support the hypothesis that KIR‐3DL2 induction occurs during a process of antigen‐driven T cell activation. Intriguingly, we also detected identical clonotypes within individuals that were shared between the KIR‐3DL2+ and KIR‐3DL2− CD4+ T cell compartments. The presence of these nucleotide‐identical sequences strongly suggests that individual T cell clones can dynamically modulate KIR‐3DL2 expression. Differential functionality has also been described for T cell clones bearing different NK cell receptors in the synovial fluid of patients with SpA, although KIR‐3DL2 was not included in that analysis 36.

KIR‐3DL2+CD4+ T cells from synovial fluid samples were further enriched for IL‐1R and OX40 expression as compared to matched samples from the peripheral blood. This finding suggests that activation‐induced KIR‐3DL2 expression could promote the survival of CD4+ T cells expressing IL‐1R and IL‐23R at sites of inflammation. The expression of OX40 by synovial KIR‐3DL2+CD4+ T cells further supports our hypothesis that antigen‐induced activation is a disease‐relevant process in SpA. Consistent with this interpretation, we observed oligoclonal expansions in the synovial KIR‐3DL2+CD4+ T cell subset in some patients with SpA. Trafficking of KIR‐3DL2+CD4+ T cells between sites of inflammation and the periphery is supported by our observation of shared clonotypes in matched peripheral blood and synovial fluid KIR‐3DL2+CD4+ T cells isolated from 2 patients with SpA. Moreover, the fact that KIR‐3DL2+CD4+ T cells were enriched for CCR9 expression suggests a possible intestinal origin. It is notable in this regard that up to 70% of patients with AS exhibit subclinical terminal ileitis 37.

Despite the fact that up to 94% of individuals with AS express B27 1, the mechanistic link that underlies this HLA‐associated disease remains unexplained. Our data support a 2‐step model, whereby T cell activation induces KIR‐3DL2 expression, and subsequent interaction with B27 free heavy chains promotes Th17 cell differentiation and survival. The same process may also occur on NK cells and CD8+ T cells, both of which can express KIR‐3DL2. In this scenario, the observed HLA linkage could be explained by the fact that KIR‐3DL2 binds more strongly to B27 dimers and free heavy chains compared to other HLA class I heavy chains 9, 38. It is likely that by priming T cells to produce IL‐17, this mechanism could act in concert with other mechanisms that have been proposed to enhance Th17 cytokine production in SpA, such as promotion of the unfolded protein response by B27 39. Collectively, these findings suggest that targeting HLA–B27–KIR‐3DL2 interactions could be therapeutically beneficial in patients with SpA.

## AUTHOR CONTRIBUTIONS

All authors were involved in drafting the article or revising it critically for important intellectual content, and all authors approved the final version for publication. Dr. Kollnberger had full access to all of the data in the study and takes responsibility for the integrity of the data and the accuracy of the data analysis.

**Study conception and design.** Ridley, Hatano, Wong‐Baeza, Shaw, Matthews, Al‐Mossawi, Ladell, Price, Bowness, Kollnberger.

### Acquisition of data

Ridley, Hatano, Wong‐Baeza, Shaw, Matthews, Al‐Mossawi, Kollnberger.

### Analysis and interpretation of data

Ridley, Hatano, Wong‐Baeza, Shaw, Matthews, Al‐Mossawi, Ladell, Price, Bowness, Kollnberger.

## Supporting information


**Supplementary Figure 1. Purity and KIR‐3DL2 expression of Miltenyi bead–sorted memory (CD45RO+CD4+) and naive (CD45RO‐CD4+) T cells.** FACS staining of a healthy control (HC; top left panel) and an AS patient (top right panel) showing that purity of bead‐sorted CD45RO‐CD4+ T cells is >96%. FACS staining of a HC shows that KIR‐3DL2+CD4+ T cells are located within the memory CD4+ T cell population (bottom left panel) but not the naive CD4+ T cell population (bottom middle), as compared to isotype control staining (bottom right panel).
**Supplementary Figure 2. Purity of KIR‐3DL2+ and KIR‐3DL2‐ CD4+ T cells after FACS sorting. Upper panels.** FACS staining showing KIR‐3DL2 expression after FACS sorting on KIR‐3DL2‐ (left) and KIR‐3DL2+ CD4+ T cells (middle), and the same data shown as a histogram (right), with KIR‐3DL2‐ (blue) and KIR‐3DL2+ (red) CD4+ T cells. **Lower panel**. qPCR analysis of mRNA from FACS‐sorted KIR‐3DL2+CD4+ T cells and KIR‐3DL2‐CD4+ T cells from peripheral blood of an HLA–B27‐ healthy control (HC) and HLA–B27+ AS patient.
**Supplementary Figure 3.** KIR‐3DL2 expression relative to day 0 detected by qPCR after 24, 72 and 120 hours of coculture of naive CD4 T cells with SEB and irradiated LBL.721.220 HLA–B27+ or LBL.721.220 HLA–B7+ cells.
**Supplementary Figure 4. KIR‐3DL2 expression, induced by naive CD4+ T cells from a B27‐ healthy control, is greater after coculture with LBL.721.220 HLA–B27+ compared to LBL.721.220 HLA–B7+ cells. A,** Naive CD4+ T cells isolated from the peripheral blood of a healthy control were cultured in the presence of LBL.721.220 HLA–B7+ or HLA–B27+ irradiated APCs and SEB. FACS staining with the anti‐KIR‐3DL2 mAb (DX31) or isotype control mAb (IgG2a) of CD45RO+ CD4+ T cells after 5 days. The histogram shows KIR‐3DL2 expression; the light‐grey line shows isotype control staining, and the grey and black lines showing KIR‐3DL2 expression after coculture with LBL.721.220 HLA–B7+ or HLA–B27+ cells, respectively. **B,** KIR‐3DL2 expression of naive T cells activated for 8 days with SEB and LBL.721.220 HLA–B27+ cells in the presence of the indicated antibodies. Representative stain from 1 of 3 independent experiments.
**Supplementary Figure 5. IL17 secretion by CD4 T cells stimulated with SEB and LBL.721.221 HLA–B27+ cells is inhibited by DX31 and HC10 antibodies.** Cells cultured in the presence of the anti–KIR‐3DL2 (DX31) (**A)** or HLA class I heavy chain antibodies (HC10) (**B).** Each point represents IL‐17 secretion by T cells from a different healthy control**. C.** IL‐17 secretion by naive T cells stimulated with anti‐CD3, anti‐CD28 and anti‐CD2 beads or LBL.721.220, LBL.721.220 HLA–B7+, and LBL.721.220 HLA–B27+ cells with SEB with (+) or without (‐) Th17 cytokines for 8 days. **D.** IL‐17 secretion by naive T cells stimulated with LBL.721.220 HLA–B27+ cells and SEB with or without Th17 cytokines in the presence of the indicated antibodies. Results in **C** and **D** are mean ± SEM values from three independent experiments. * *P* < 0.05, **P < 0.01, ***P < 0.005, comparing LBL.721.220 HLA–B27 and other stimuli in **C** by ANOVA and LBL.721.220 HLA–B27+ IgG2a with LBL.721.220 HLA–B27 + HC10 and LBL.721.220 HLA–B27 + DX31 using Student's *t*‐test.
**Supplementary Figure 6. Targeting KIR‐3DL2 B27 heavy chain interactions inhibits IL‐17 production by differentiating Th17 cells**. **A**.Representative FACS staining of IL‐17 and IFNγ production by naive CD4 T cells stimulated for 8 days with SEB,Th17 cytokines and LBL.721.220 or LBL.721.220 HLA–B7+ cells (upper panels) or LBL.721.220 HLA–B27+ cells with isotype control (IgG2a) or anti‐class I heavy chain (HC10) or anti–KIR‐3DL2 (DX31) antibodies (lower panels). **B.** Representative FACS stain of KIR‐3DL2 expression by IL‐17+ T cells following 8 day stimulation with SEB, Th17 cytokines and LBL.721.220 HLA–B27+ cells in the presence of the indicated antibodies. FACS stains are representative of 1 of 4 independent experiments. The total numbers of IL‐17+ and IFN+ T cells counted in each sample are indicated. **C.** Proportions of IL‐17+ CD4 T cells expressed as percentage of Th17 cells stimulated with LBL.721.220 HLA–B27+ cells following 8 day stimulation of naive T cells with SEB and Th17 cytokines with anti‐CD3/CD2/CD28 beads or the indicated cell lines. Data are mean ± SEM from four independent experiments. **D.** Numbers of IL‐17+T cells/100,000 total antigen presenting cells and T cells following stimulation of naive T cells with Th17 cytokines and 220B27 cells in the presence of the indicated antibodies. Data are mean ± SEM from 3 independent experiments. *P < 0.05, unpaired Student's *t*‐test.
**Supplementary Figure 7. Effects of HLA–B27+ cells and blocking antibodies on IL‐17 production by naive T cells differentiated with superantigen and Th17 cytokines. A.** Effect of HLA–B27+ B cell lines (BCL) compared to activation with HLA–B27‐ BCL. Supernatants from 8 day activation were assayed for IL‐17 production. Results are the mean ± SEM from naive T cells isolated from two individuals activated with 3 HLA–B27+ BCL compared to activation with 4 HLA–B27‐ BCL. * indicates *P* < 0.05 by Student's *t*‐test. **B** Effect of HLA class I heavy chain (HC10) and anti–KIR‐3DL2 (DX31) and isotype control (IgG2a) antibodies on IL‐17 production by naive T cells stimulated with HLA–B27+ BCL. Results are the mean ± SEM from two independent experiments with naive T cells from two individuals stimulated with a HLA–B27+ B cell line. IL‐17 production with HC10 and DX31 antibodies was significantly lower than production with isotype control antibody (*P* < 0.01 and *P* < 0.005, by Student's *t*‐test)
**Supplementary Figure 8.** HD6 (anti‐B27 dimer) and DX31 (anti–KIR‐3DL2) antibodies inhibit survival and proliferation of antigen activated CFSE‐labeled KIR‐3DL2 CD4 T cells from the PBMCs of an HLA–B27–positive SpA patient but not an HLA–B27–negative healthy control. Representative stain from 1 of 3 independent experiments.
**Supplementary Figure 9. KIR‐3DL2+ sorted CD4+ T cells are enriched for CCR6 and IL‐23R mRNA**. **Upper panels.** qPCR analysis of mRNA from FACS sorted KIR‐3DL2+ and KIR‐3DL2‐ CD4+ T cells from peripheral blood of an AS patient and an HLA–B27‐ healthy control showing expression of IL‐23R (left) and CCR6 (right), normalized to CD3. Bars represent mean ± SD. Data was analyzed using the ΔΔC_t_ method. **Lower panel.** IL‐6 secretion by FACS sorted KIR‐3DL2+ and KIR‐3DL2‐ CD4 T cells from peripheral blood samples from 7 AS patients.

Supplementary Table 1. Patients and healthy control subjects in this study
**Supplementary Table 2.** TCR CDR3 sequences and TRBV and J gene usage for the ankylosing spondylitis (AS) and B27‐ healthy control subjects in this study. Sequences which are identical in KIR‐3DL2+ and KIR‐3DL2‐ sorted fractions are colored similarly.
