# Supplementary material for: Activation‐Induced Killer Cell Immunoglobulin‐like Receptor 3DL2 Binding to HLA–B27 Licenses Pathogenic T Cell Differentiation in Spondyloarthritis
Source: Arthritis Rheumatol. 2016 Mar 28;68(4):901–14. doi: 10.1002/art.39515 (PMC4855641; doi:10.1002/art.39515)

**Supplementary Figure 1. Purity and KIR-3DL2 expression of Miltenyi bead–sorted memory (CD45RO+CD4+) and naive (CD45RO-CD4+) T cells.** FACS staining of a healthy control (HC; top left panel) and an AS patient (top right panel) showing that purity of bead-sorted CD45RO-CD4+ T cells is >96%. FACS staining of a HC shows that KIR-3DL2+CD4+ T cells are located within the memory CD4+ T cell population (bottom left panel) but not the naive CD4+ T cell population (bottom middle), as compared to isotype control staining (bottom right panel).

**
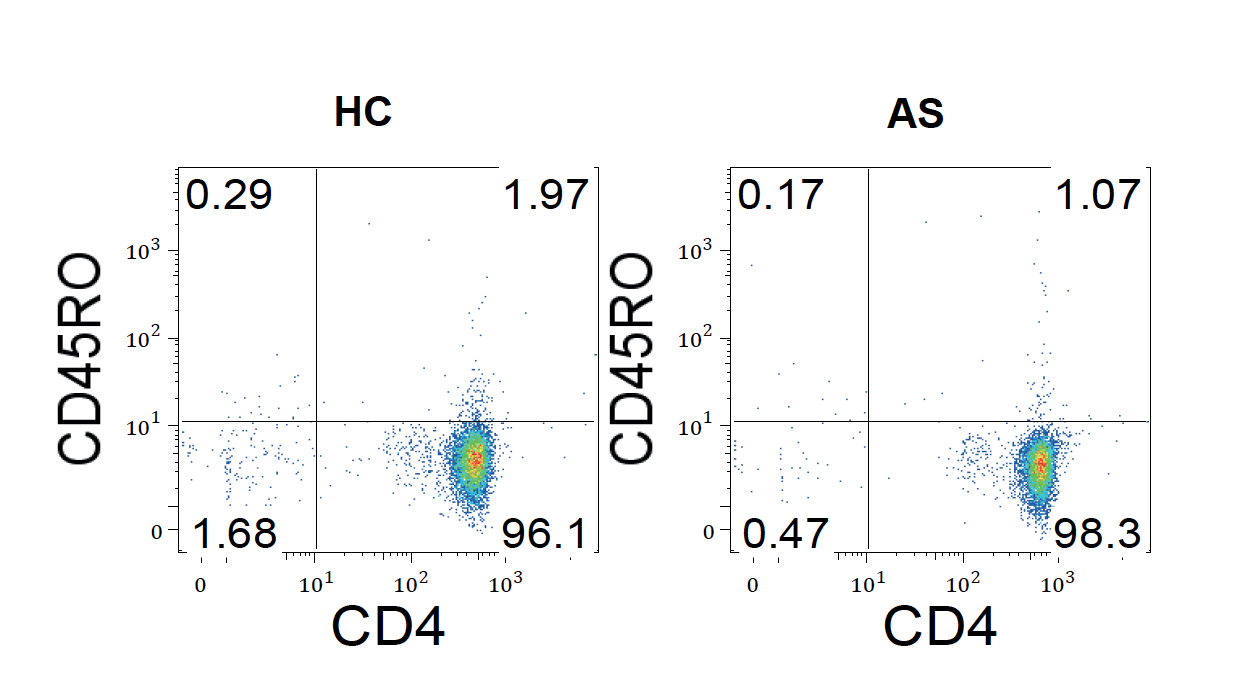
**

**
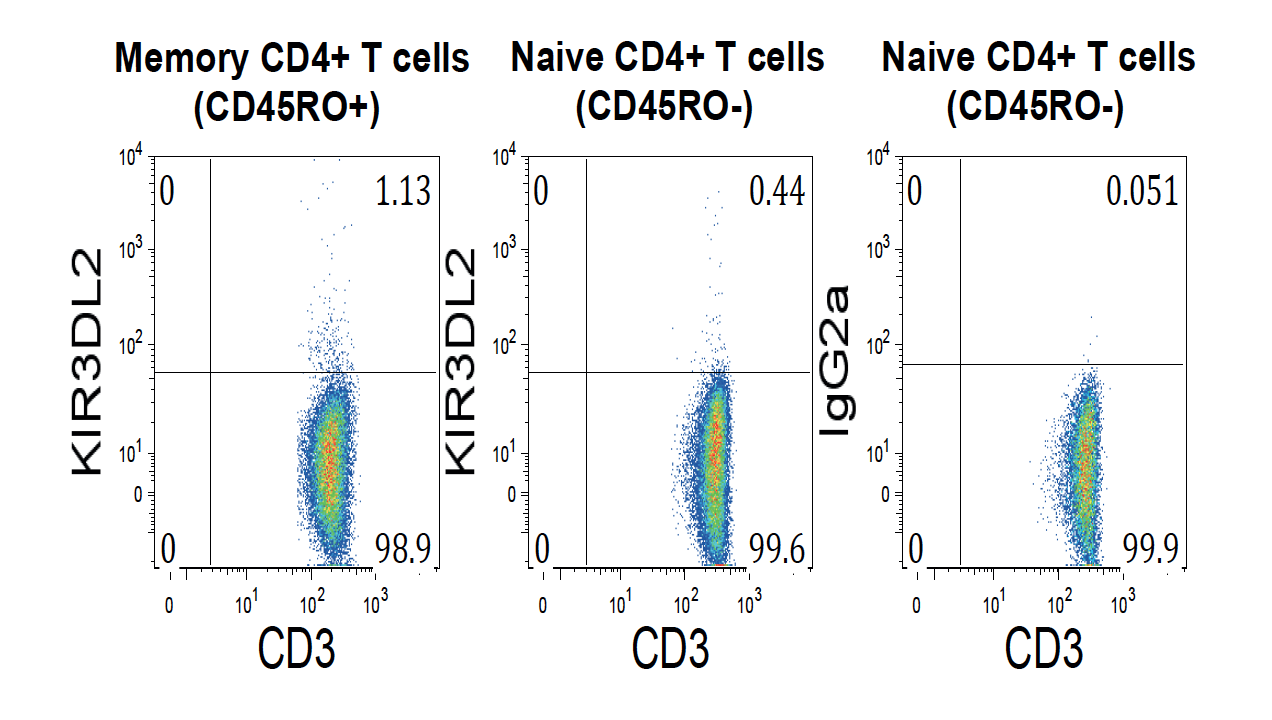
**

**Supplementary Figure 2. Purity of KIR-3DL2+ and KIR-3DL2- CD4+ T cells after FACS sorting. Upper panels.** FACS staining showing KIR-3DL2 expression after FACS sorting on KIR-3DL2- (left) and KIR-3DL2+ CD4+ T cells (middle), and the same data shown as a histogram (right), with KIR-3DL2- (blue) and KIR-3DL2+ (red) CD4+ T cells. **Lower panel**. qPCR analysis of mRNA from FACS-sorted KIR-3DL2+CD4+ T cells and KIR-3DL2-CD4+ T cells from peripheral blood of an HLA–B27- healthy control (HC) and HLA–B27+ AS patient.

**
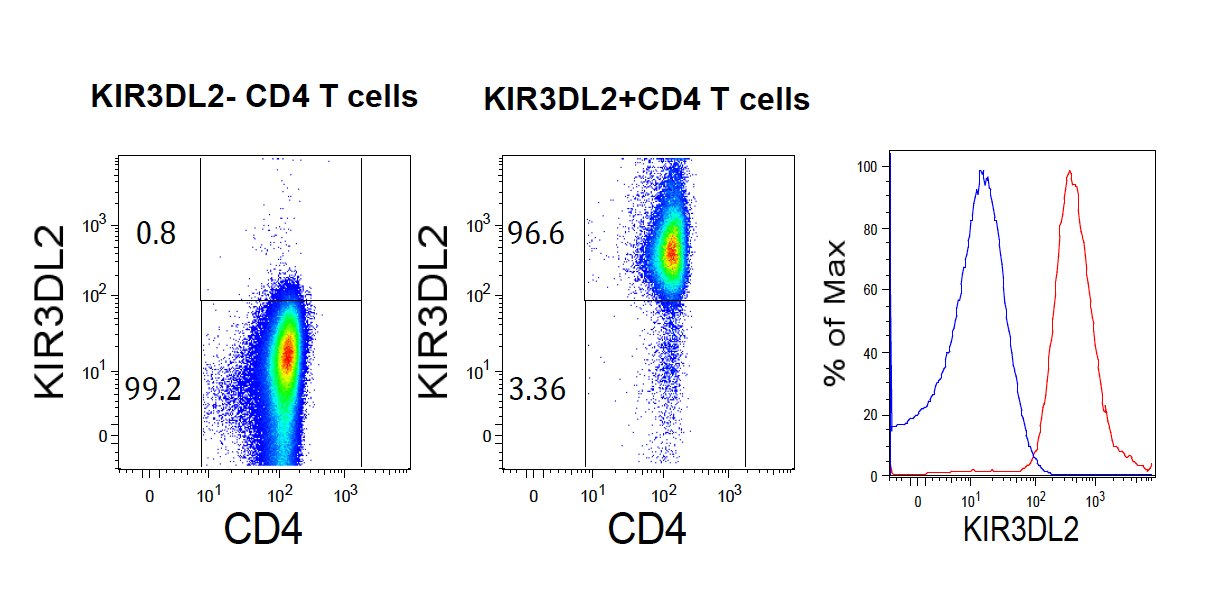

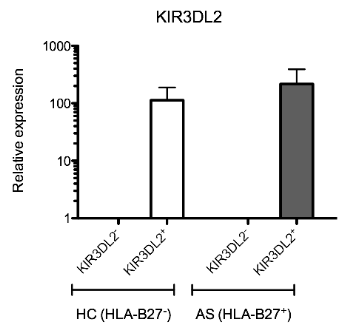
**

**Supplementary Figure 3.** KIR-3DL2 expression relative to day 0 detected by qPCR after 24, 72 and 120 hours of coculture of naive CD4 T cells with SEB and irradiated LBL.721.220 HLA–B27+ or LBL.721.220 HLA–B7+ cells.

**
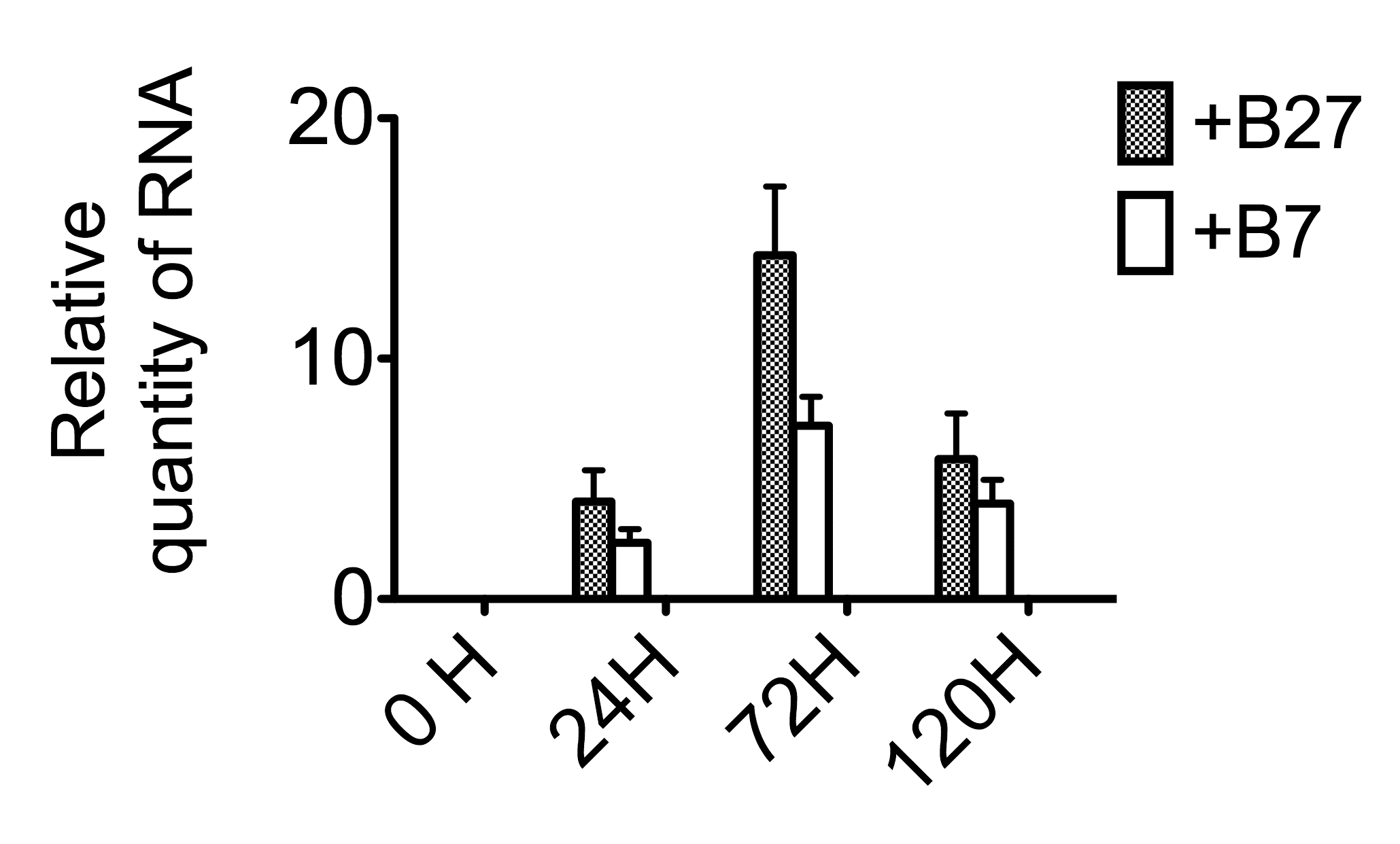
**

**Supplementary Figure 4. KIR-3DL2 expression, induced by naive CD4+ T cells from a B27- healthy control, is greater after coculture with LBL.721.220 HLA–B27+ compared to LBL.721.220 HLA–B7+ cells. A,** Naive CD4+ T cells isolated from the peripheral blood of a healthy control were cultured in the presence of LBL.721.220 HLA–B7+ or HLA–B27+ irradiated APCs and SEB. FACS staining with the anti-KIR-3DL2 mAb (DX31) or isotype control mAb (IgG2a) of CD45RO+ CD4+ T cells after 5 days. The histogram shows KIR-3DL2 expression; the light-grey line shows isotype control staining, and the grey and black lines showing KIR-3DL2 expression after coculture with LBL.721.220 HLA–B7+ or HLA–B27+ cells, respectively. **B,** KIR-3DL2 expression of naive T cells activated for 8 days with SEB and LBL.721.220 HLA–B27+ cells in the presence of the indicated antibodies. Representative stain from 1 of 3 independent experiments.

**
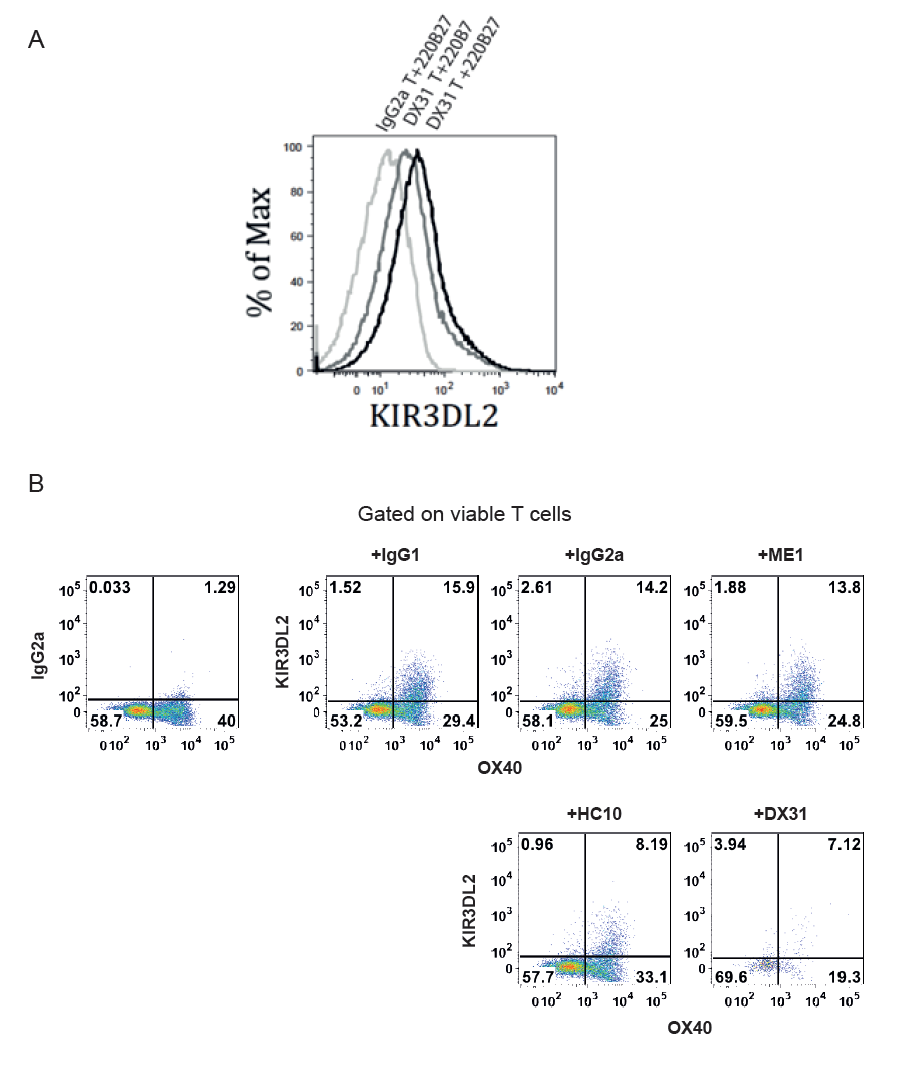
**

**Supplementary Figure 5. IL17 secretion by CD4 T cells stimulated with SEB and LBL.721.221 HLA–B27+ cells is inhibited by DX31 and HC10 antibodies.** Cells cultured in the presence of the anti–KIR-3DL2 (DX31) (**A)** or HLA class I heavy chain antibodies (HC10) (**B).** Each point represents IL-17 secretion by T cells from a different healthy control**. C.** IL-17 secretion by naive T cells stimulated with anti-CD3, anti-CD28 and anti-CD2 beads or LBL.721.220, LBL.721.220 HLA–B7+, and LBL.721.220 HLA–B27+ cells with SEB with (+) or without (-) Th17 cytokines for 8 days. **D.** IL-17 secretion by naive T cells stimulated with LBL.721.220 HLA–B27+ cells and SEB with or without Th17 cytokines in the presence of the indicated antibodies. Results in **C** and **D** are mean ± SEM values from three independent experiments. * P <0.05, **P<0.01, ***P<0.005, comparing LBL.721.220 HLA–B27 and other stimuli in **C** by ANOVA and LBL.721.220 HLA–B27+ IgG2a with LBL.721.220 HLA–B27 + HC10 and LBL.721.220 HLA–B27 + DX31 using Student’s *t*-test.

**A** **B**

**C D**

**Supplementary Figure 6. Targeting KIR-3DL2 B27 heavy chain interactions inhibits IL-17 production by differentiating Th17 cells**. **A**.Representative FACS staining of IL-17 and IFNγ production by naive CD4 T cells stimulated for 8 days with SEB,Th17 cytokines and LBL.721.220 or LBL.721.220 HLA–B7+ cells (upper panels) or LBL.721.220 HLA–B27+ cells with isotype control (IgG2a) or anti-class I heavy chain (HC10) or anti–KIR-3DL2 (DX31) antibodies (lower panels). **B.** Representative FACS stain of KIR-3DL2 expression by IL-17+ T cells following 8 day stimulation with SEB, Th17 cytokines and LBL.721.220 HLA–B27+ cells in the presence of the indicated antibodies. FACS stains are representative of 1 of 4 independent experiments. The total numbers of IL-17+ and IFN+ T cells counted in each sample are indicated. **C.** Proportions of IL-17+ CD4 T cells expressed as percentage of Th17 cells stimulated with LBL.721.220 HLA–B27+ cells following 8 day stimulation of naive T cells with SEB and Th17 cytokines with anti-CD3/CD2/CD28 beads or the indicated cell lines. Data are mean ± SEM from four independent experiments. **D.** Numbers of IL-17+T cells /100,000 total antigen presenting cells and T cells following stimulation of naive T cells with Th17 cytokines and 220B27 cells in the presence of the indicated antibodies. Data are mean ± SEM from 3 independent experiments. *P<0.05, unpaired Student’s *t*-test.

**
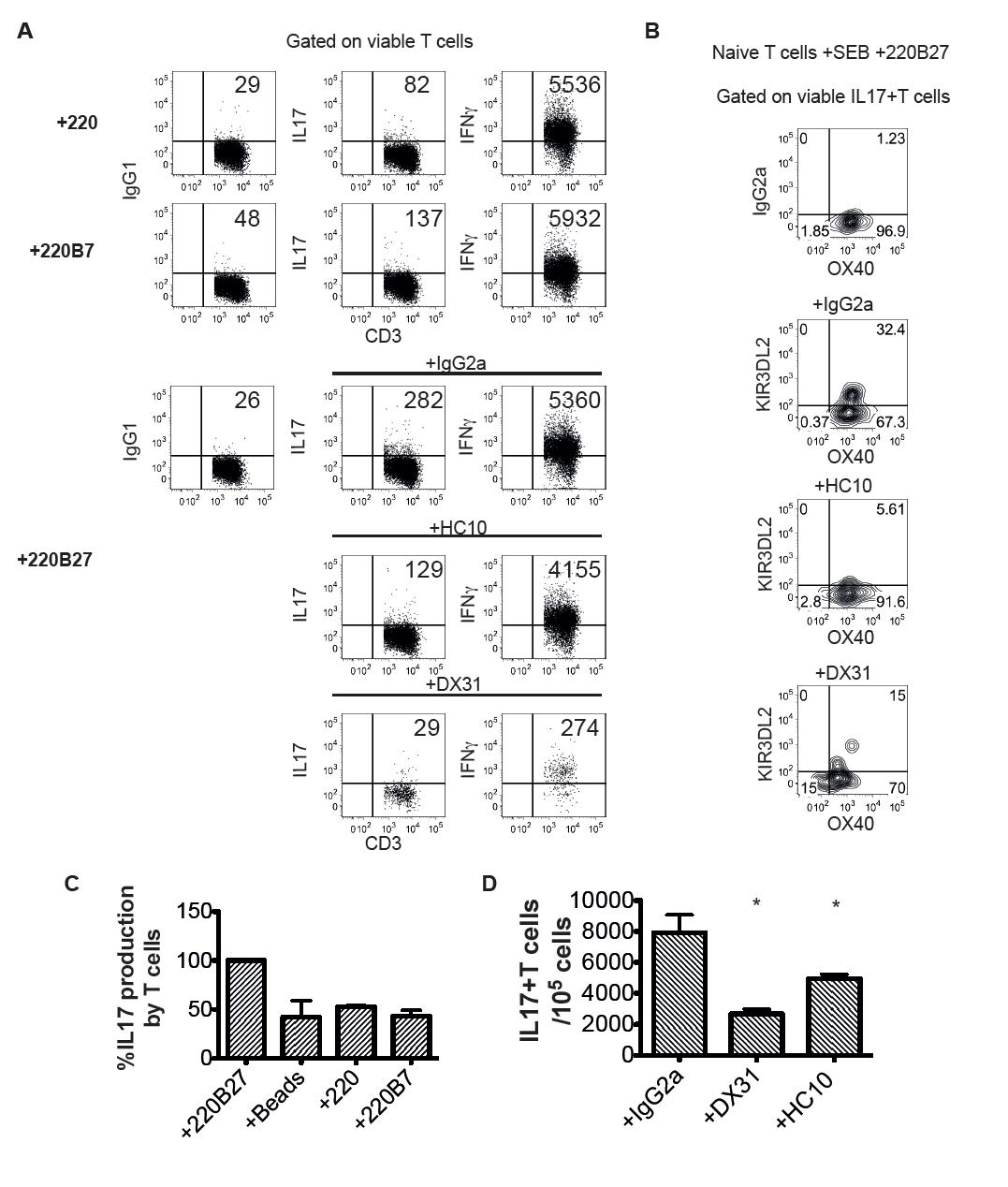
**

**Supplementary Figure 7. Effects of HLA–B27+ cells and blocking antibodies on IL-17** **production by naive T cells differentiated with superantigen and Th17 cytokines. A.** Effect of HLA–B27+ B cell lines (BCL) compared to activation with HLA–B27- BCL. Supernatants from 8 day activation were assayed for IL-17 production. Results are the mean ± SEM from naive T cells isolated from two individuals activated with 3 HLA–B27+ BCL compared to activation with 4 HLA–B27- BCL. * indicates P<0.05 by Student’s *t*-test. **B** Effect of HLA class I heavy chain (HC10) and anti–KIR-3DL2 (DX31) and isotype control (IgG2a) antibodies on IL-17 production by naive T cells stimulated with HLA–B27+ BCL. Results are the mean ± SEM from two independent experiments with naive T cells from two individuals stimulated with a HLA–B27+ B cell line. IL-17 production with HC10 and DX31 antibodies was significantly lower than production with isotype control antibody (P< 0.01 and P<0.005, by Student’s *t*-test)

**A**

**B**

**Supplementary Figure 8.** HD6 (anti-B27 dimer) and DX31 (anti–KIR-3DL2) antibodies inhibit survival and proliferation of antigen activated CFSE-labeled KIR-3DL2 CD4 T cells from the PBMCs of an HLA–B27–positive SpA patient but not an HLA–B27–negative healthy control. Representative stain from 1 of 3 independent experiments.


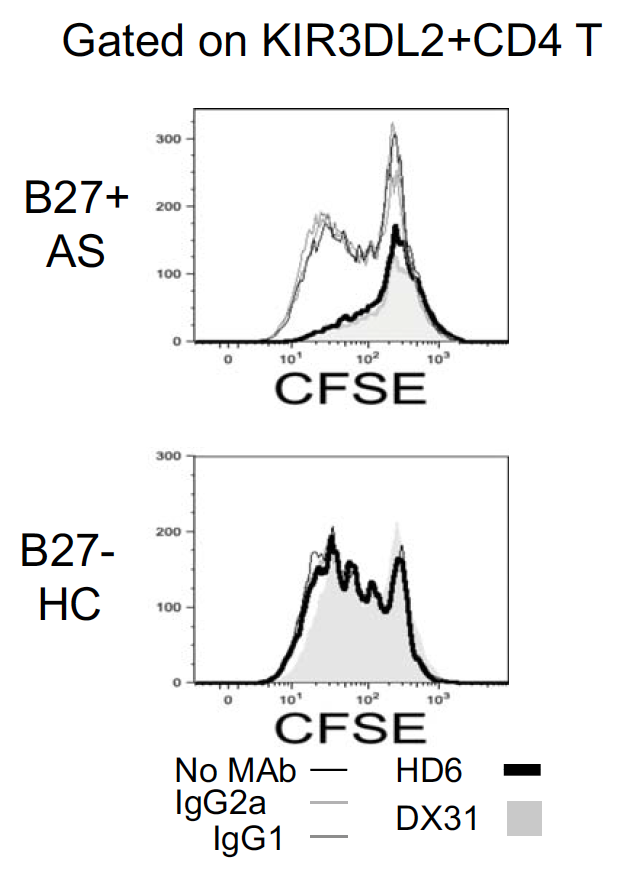


**Supplementary Figure 9. KIR-3DL2+ sorted CD4+ T cells are enriched for CCR6 and IL-23R mRNA**. **Upper panels.** qPCR analysis of mRNA from FACS sorted KIR-3DL2+ and KIR-3DL2- CD4+ T cells from peripheral blood of an AS patient and an HLA–B27- healthy control showing expression of IL-23R (left) and CCR6 (right), normalized to CD3. Bars represent mean ± SD. Data was analyzed using the ΔΔC_t_ method. **Lower panel.** IL-6 secretion by FACS sorted KIR-3DL2+ and KIR-3DL2- CD4 T cells from peripheral blood samples from 7 AS patients.


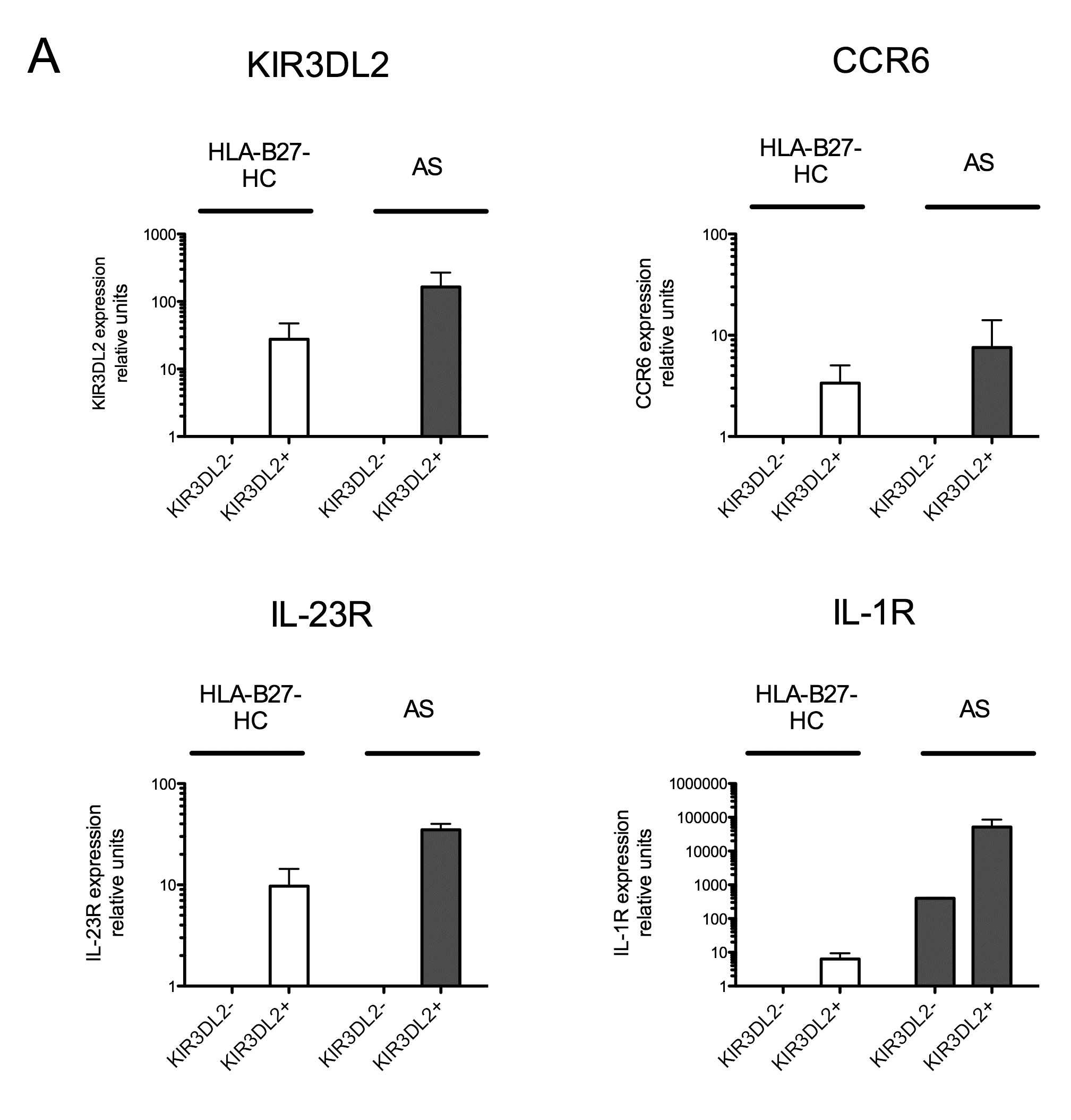


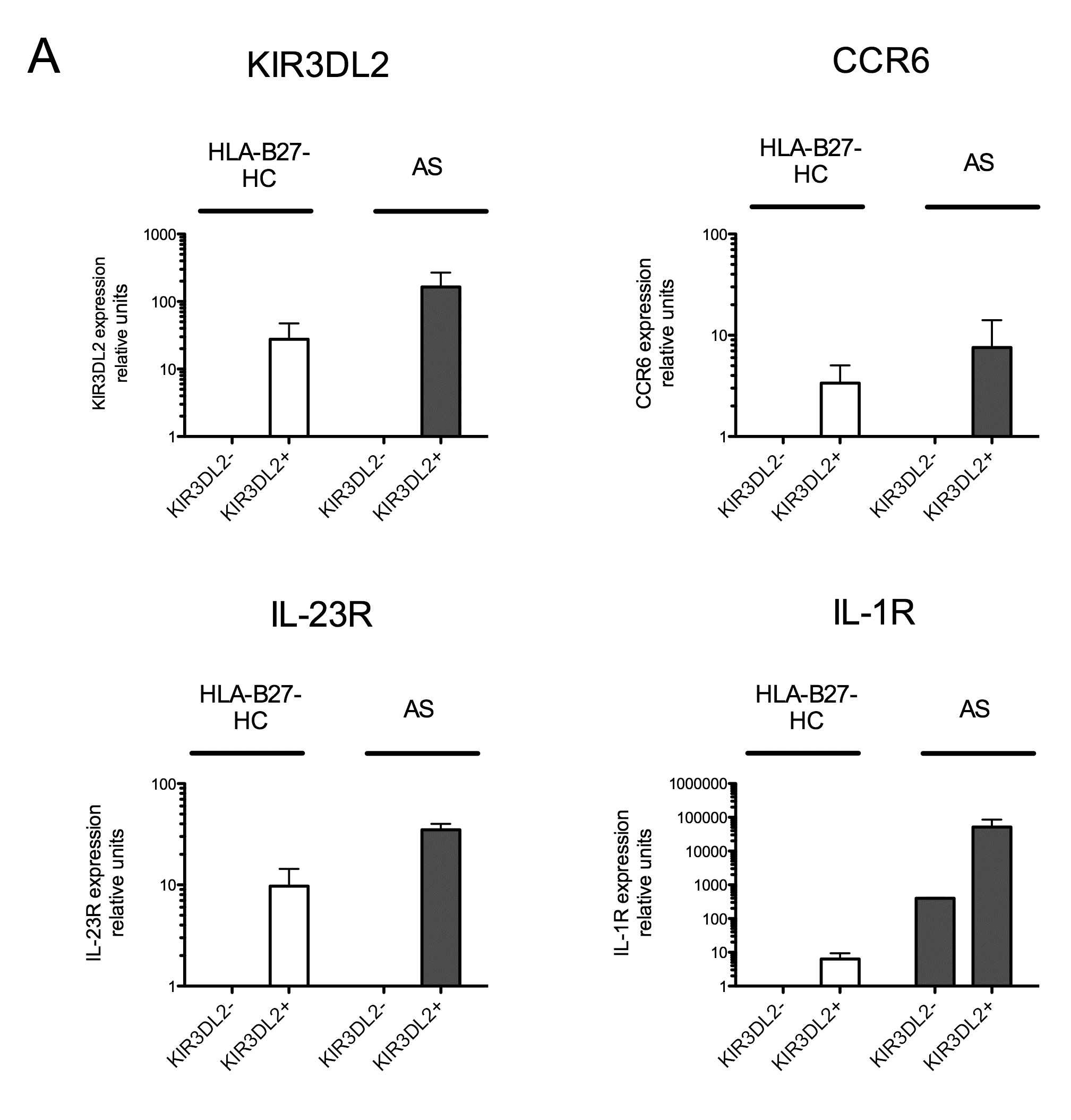


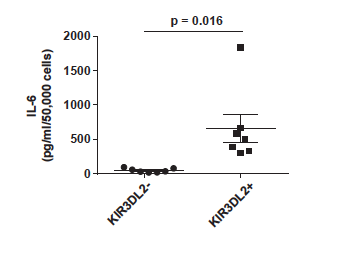

Supplement: Supplementary file 1 — Supplementary Figure 1. Purity and KIR‐3DL2 expression of Miltenyi bead–sorted memory (CD45RO+CD4+) and naive (CD45RO‐CD4+) T cells. FACS staining of a healthy control (HC; top left panel) and an AS patient (top right panel) showing that purity of bead‐sorted CD45RO‐CD4+ T cells is >96%. FACS staining of a HC shows that KIR‐3DL2+CD4+ T cells are located within the memory CD4+ T cell population (bottom left panel) but not the naive CD4+ T cell population (bottom middle), as compared to isotype control staining (bottom right panel). Supplementary Figure 2. Purity of KIR‐3DL2+ and KIR‐3DL2‐ CD4+ T cells after FACS sorting. Upper panels. FACS staining showing KIR‐3DL2 expression after FACS sorting on KIR‐3DL2‐ (left) and KIR‐3DL2+ CD4+ T cells (middle), and the same data shown as a histogram (right), with KIR‐3DL2‐ (blue) and KIR‐3DL2+ (red) CD4+ T cells. Lower panel. qPCR analysis of mRNA from FACS‐sorted KIR‐3DL2+CD4+ T cells and KIR‐3DL2‐CD4+ T cells from peripheral blood of an HLA–B27‐ healthy control (HC) and HLA–B27+ AS patient. Supplementary Figure 3. KIR‐3DL2 expression relative to day 0 detected by qPCR after 24, 72 and 120 hours of coculture of naive CD4 T cells with SEB and irradiated LBL.721.220 HLA–B27+ or LBL.721.220 HLA–B7+ cells. Supplementary Figure 4. KIR‐3DL2 expression, induced by naive CD4+ T cells from a B27‐ healthy control, is greater after coculture with LBL.721.220 HLA–B27+ compared to LBL.721.220 HLA–B7+ cells. A, Naive CD4+ T cells isolated from the peripheral blood of a healthy control were cultured in the presence of LBL.721.220 HLA–B7+ or HLA–B27+ irradiated APCs and SEB. FACS staining with the anti‐KIR‐3DL2 mAb (DX31) or isotype control mAb (IgG2a) of CD45RO+ CD4+ T cells after 5 days. The histogram shows KIR‐3DL2 expression; the light‐grey line shows isotype control staining, and the grey and black lines showing KIR‐3DL2 expression after coculture with LBL.721.220 HLA–B7+ or HLA–B27+ cells, respectively. B, KIR‐3DL2 expression of naive T [file ART-68-901-s001.doc]
