# Supplementary material for: Activation‐Induced Killer Cell Immunoglobulin‐like Receptor 3DL2 Binding to HLA–B27 Licenses Pathogenic T Cell Differentiation in Spondyloarthritis
Source: Arthritis Rheumatol. 2016 Mar 28;68(4):901–14. doi: 10.1002/art.39515 (PMC4855641; doi:10.1002/art.39515)
Supplement: Supplementary file 2 — Supplementary Table 1. Patients and healthy control subjects in this study Supplementary Table 2. TCR CDR3 sequences and TRBV and J gene usage for the ankylosing spondylitis (AS) and B27‐ healthy control subjects in this study. Sequences which are identical in KIR‐3DL2+ and KIR‐3DL2‐ sorted fractions are colored similarly. [file ART-68-901-s002.docx]

**Supplementary Table 1. Patients and healthy control subjects in this study**

| Group | Number | Age (range) | Sex  M/F | HLA-B27+ (%) | Steroids  (%) | DMARDS  (%) | Biologics  (%) |
| --- | --- | --- | --- | --- | --- | --- | --- |
| SpA ^±^ | 34 | 44  (22-66)* | 20/14 | 30  (100)^+^ | 0 | 3  (9) | 9  (27) |
| RA | 9 | 49  (24-74) | 2/7 | NT | 0 | 0 | 1  (12) |
| B27- HC | 20 | 33  (24-54) | 15/5 | 0 | 0 | 0 | 0 |
| B27+ HC | 8 | 39  (29-70) | 3/5 | 8  (100) | 0 | 0 | 0 |

^±^ Thirty one patients had AS, two had psoriatic arthritis and one had reactive arthritis

* Data available for 32 of 34 SpA patients

^+^ Data available for 28 of 34 SpA patients

**Supplementary Table 2.** TCR CDR3 sequences and TRBV and J gene usage for the ankylosing spondylitis (AS) and B27- healthy control subjects in this study. Sequences which are identical in KIR-3DL2+ and KIR-3DL2- sorted fractions are colored similarly.

**
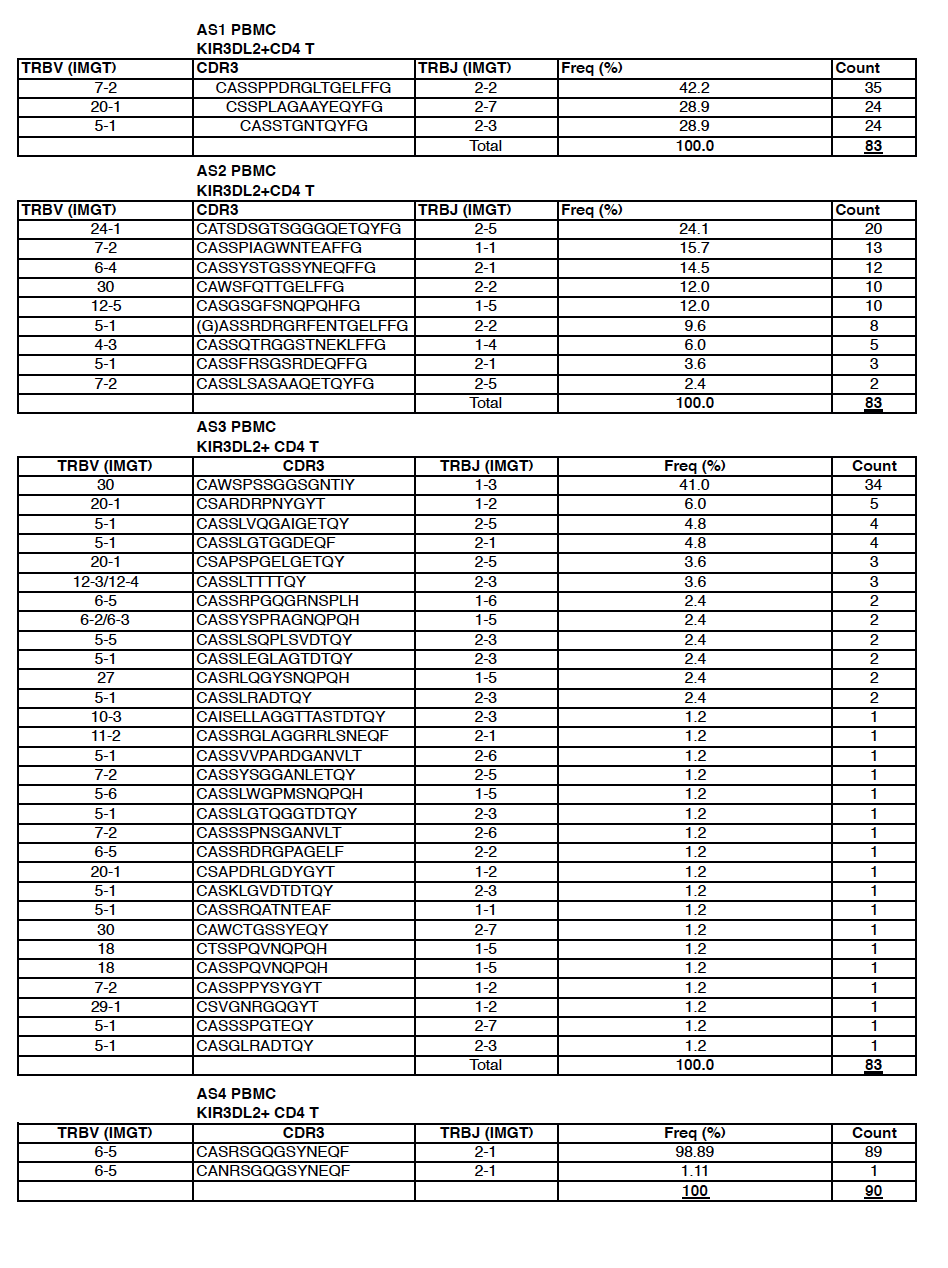
**

**
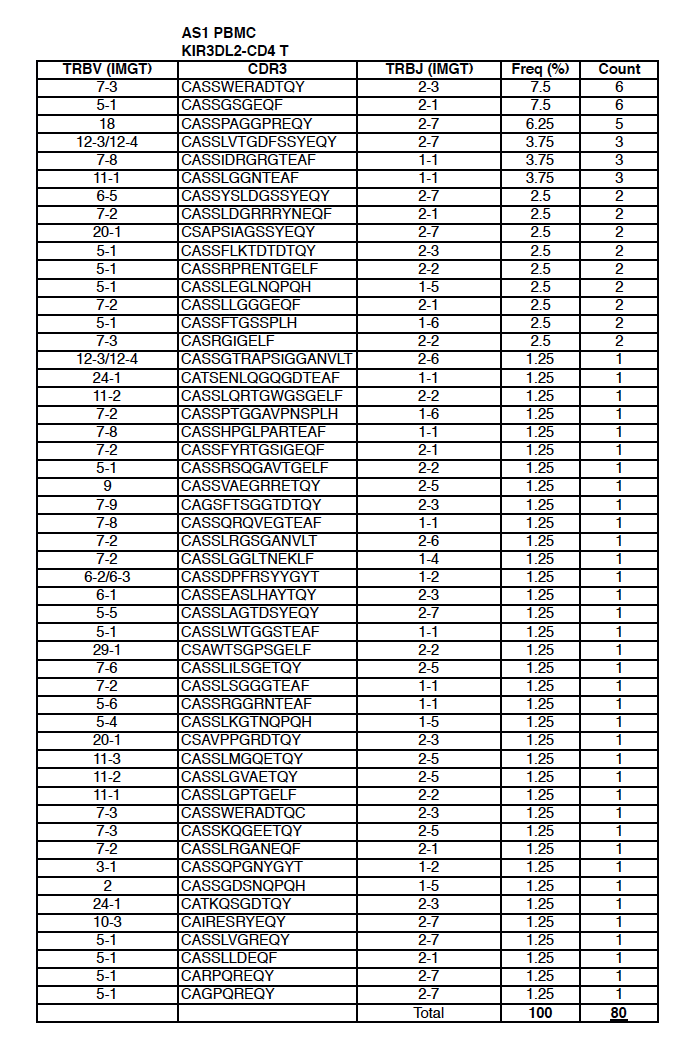
**

**
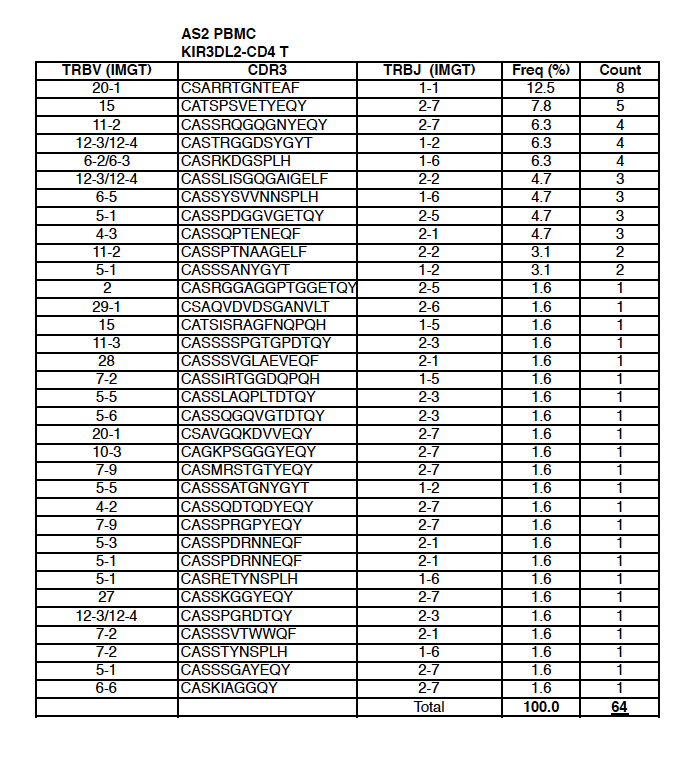
**

**
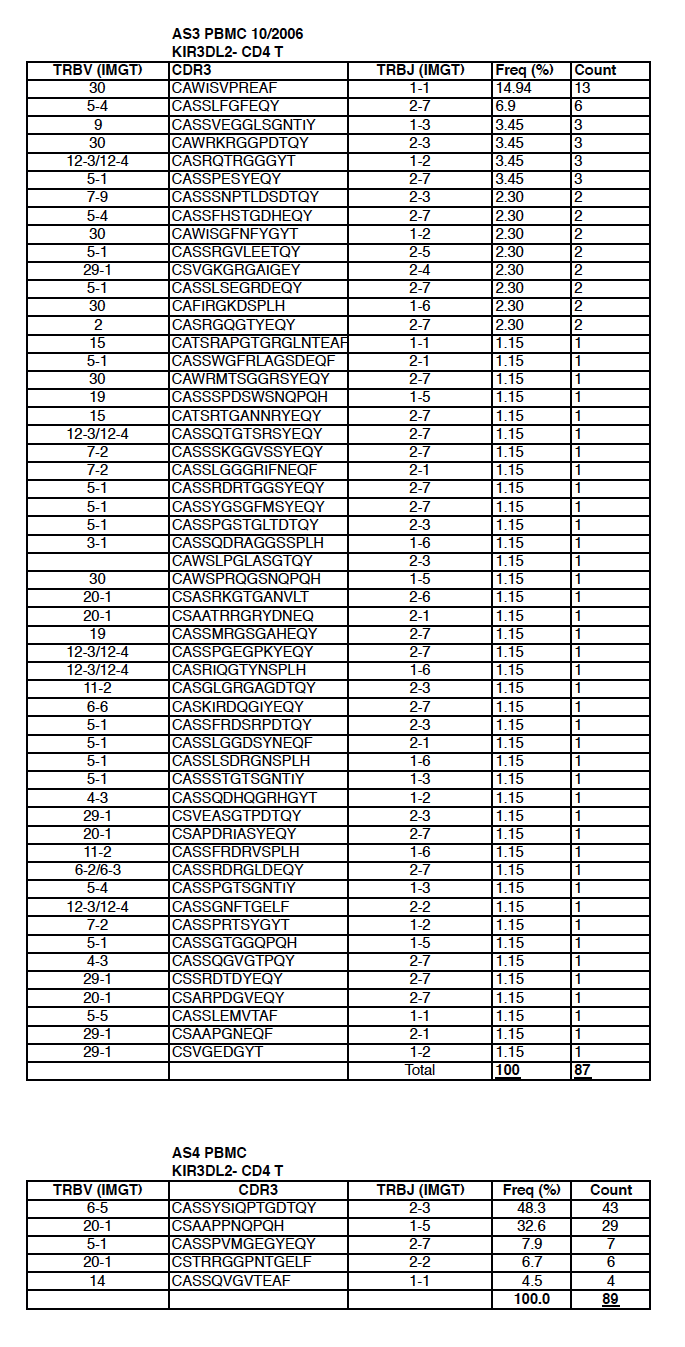
**

**
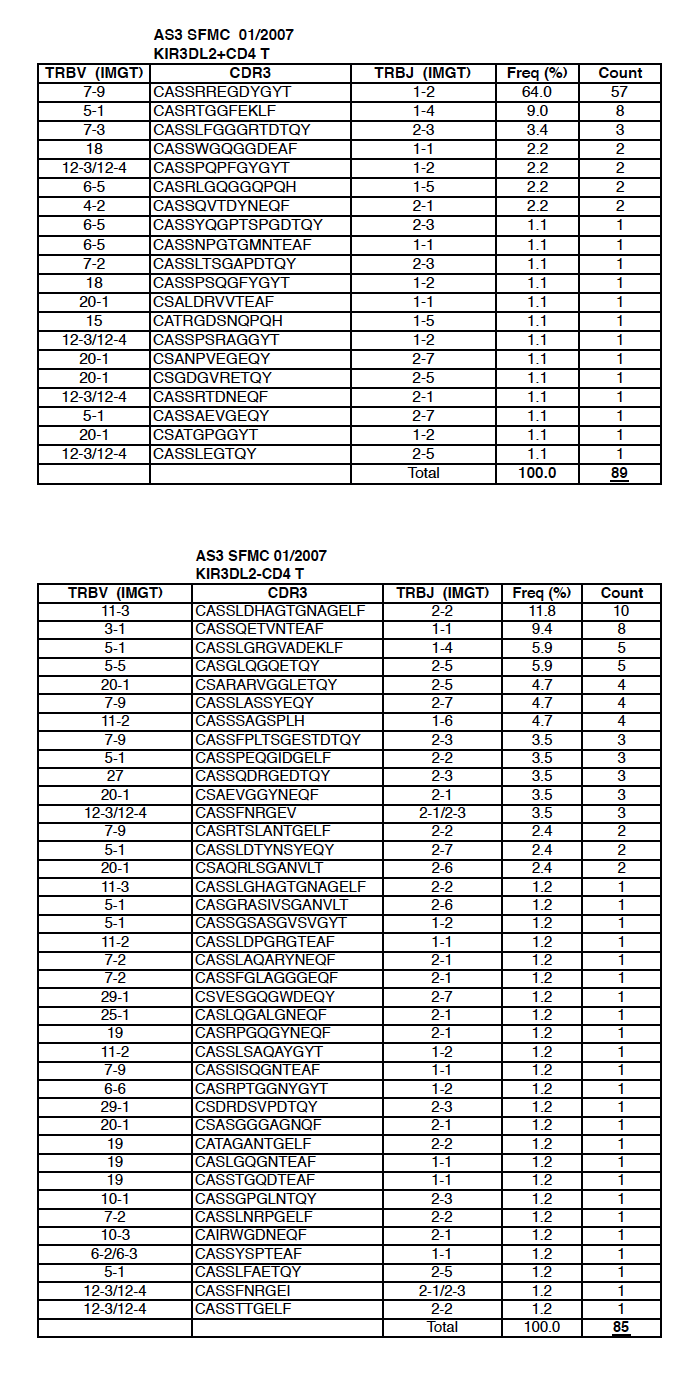
**

**
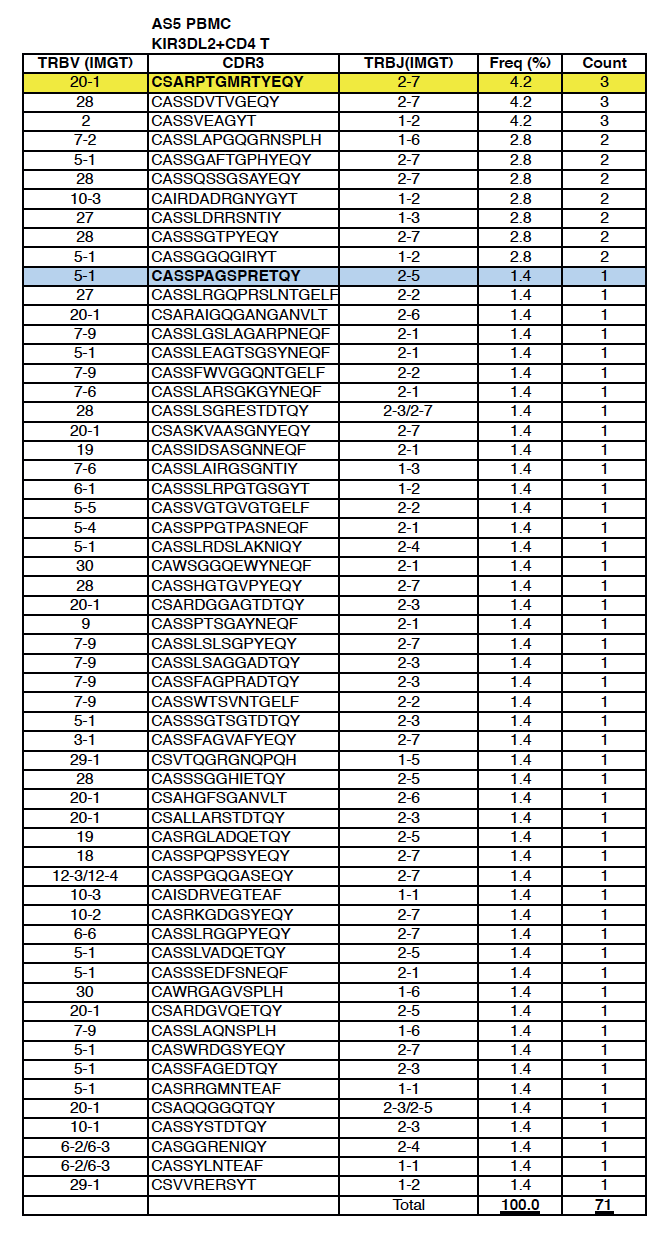
**

**
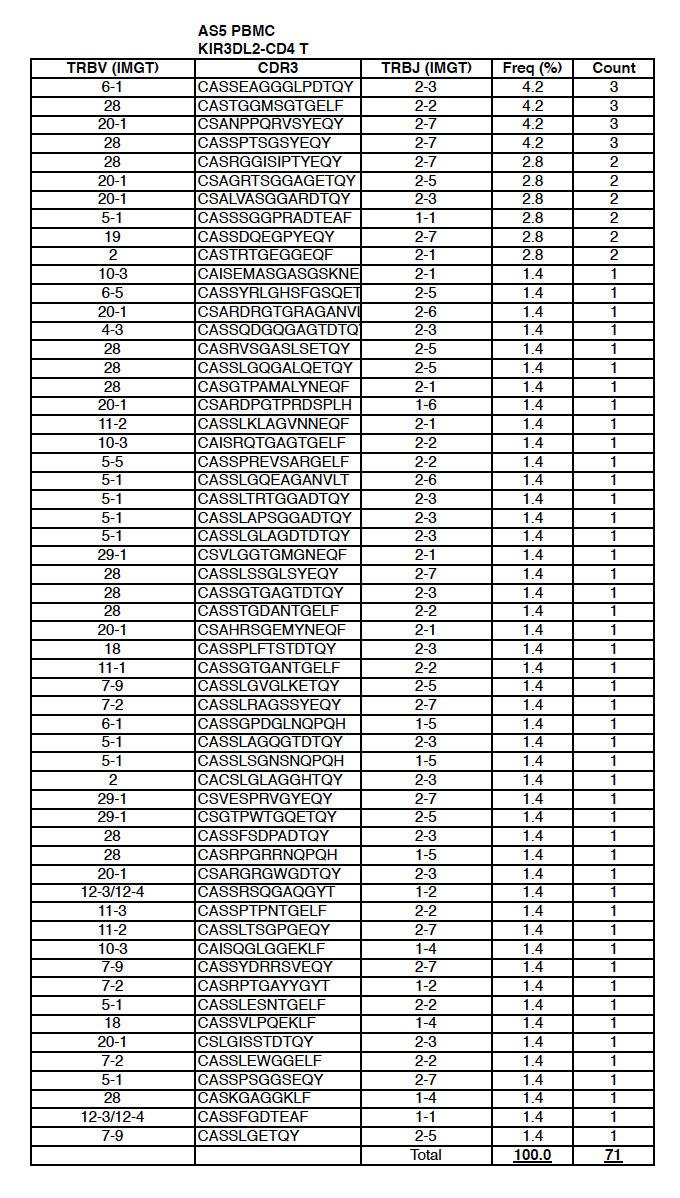
**

**
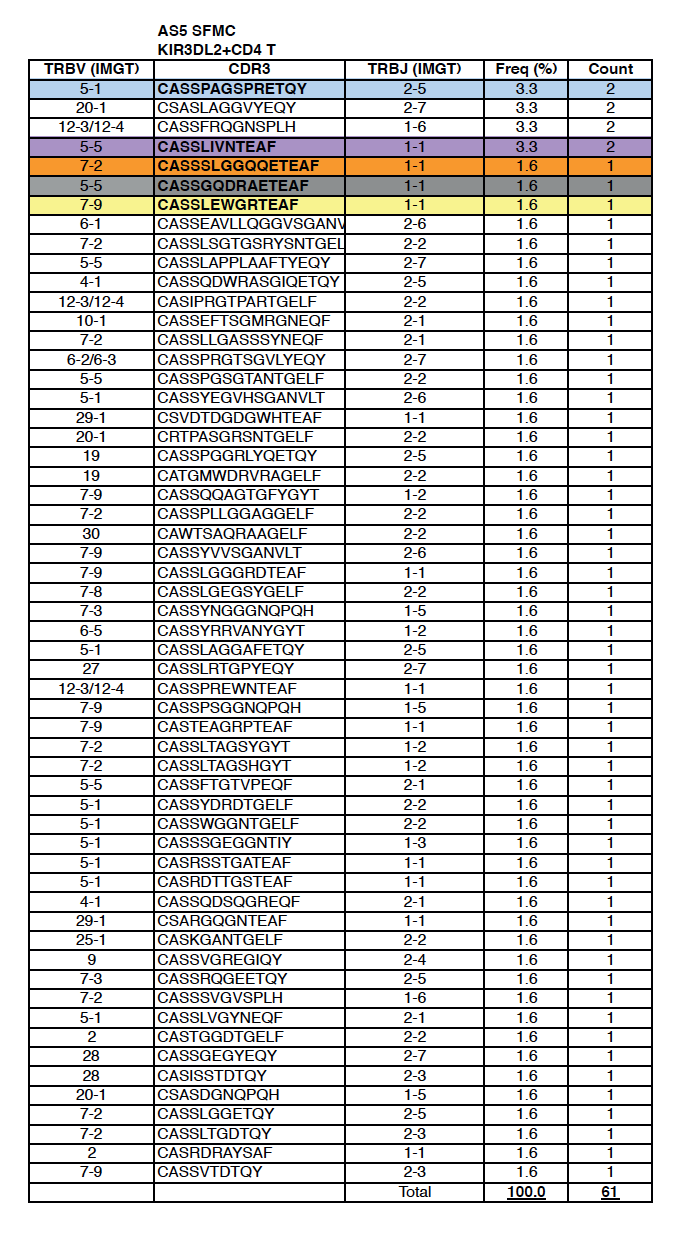
**

**
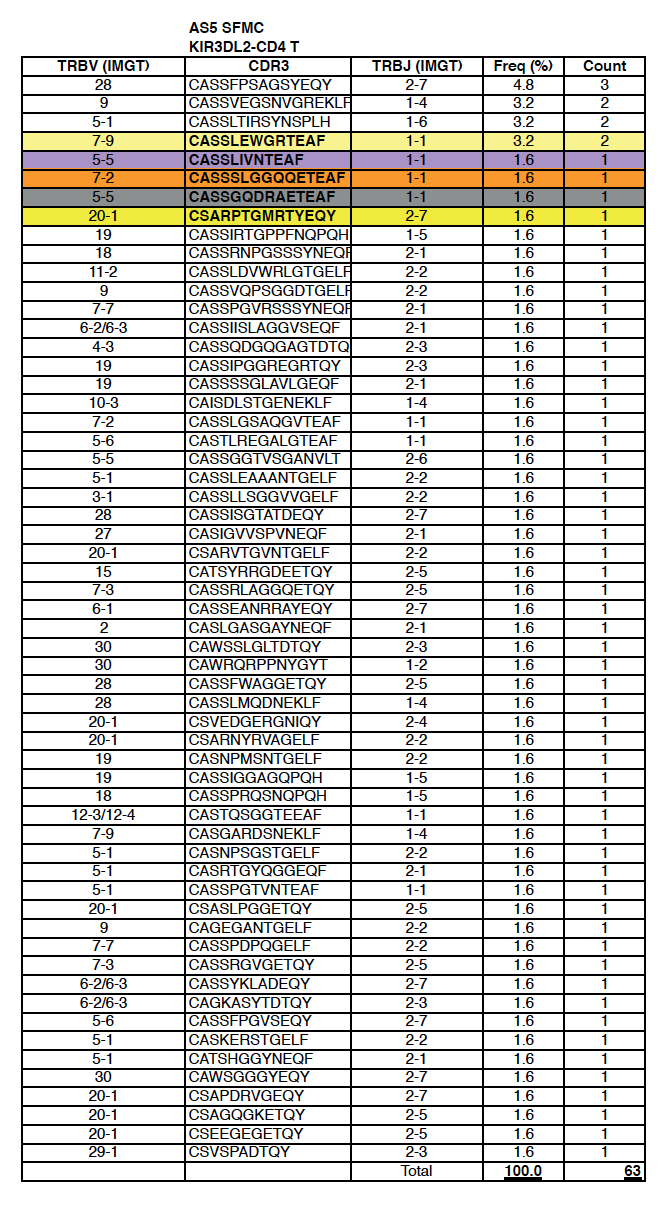
**

**
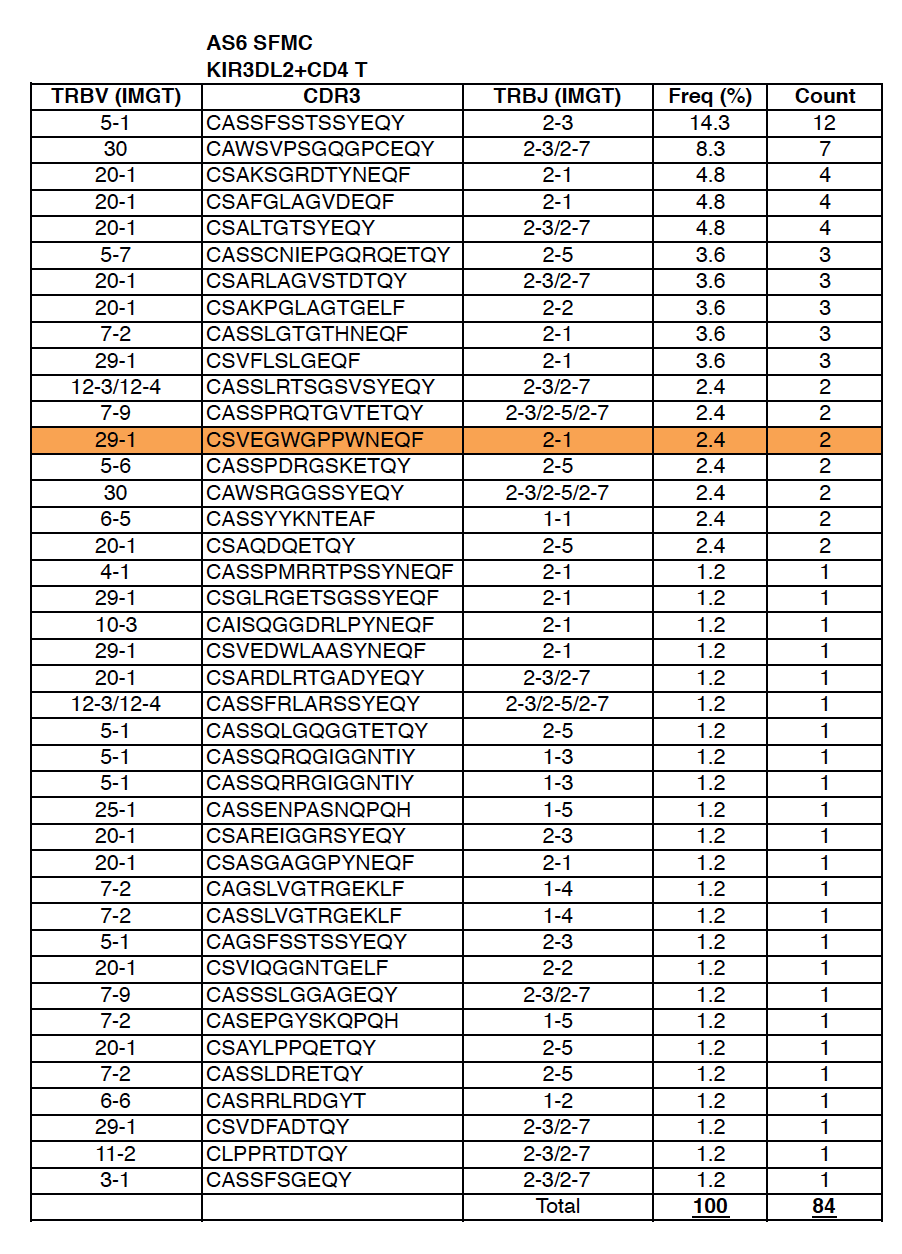
**

**
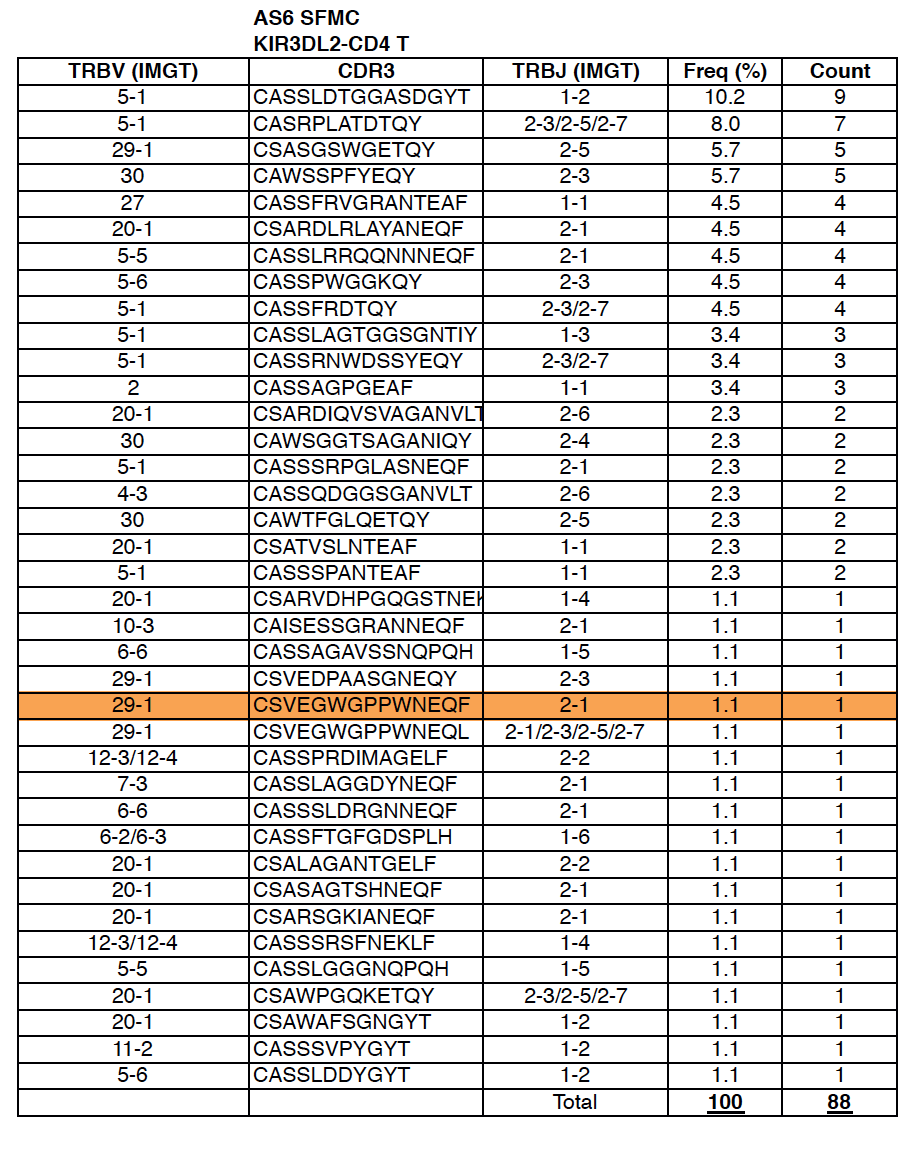
**

**
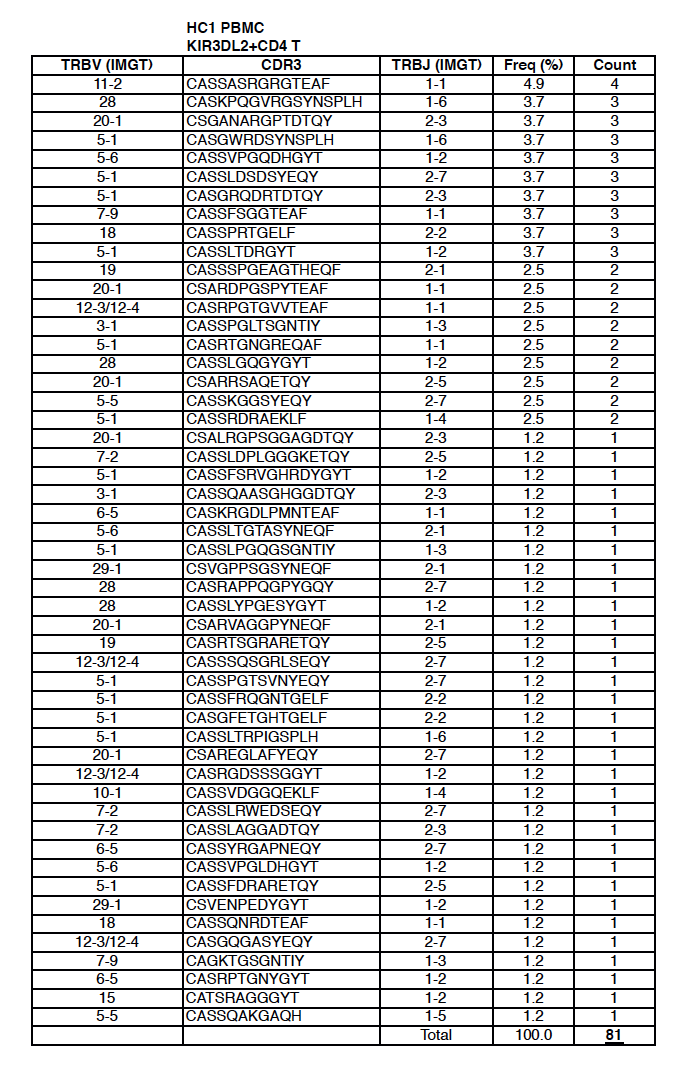
**

**
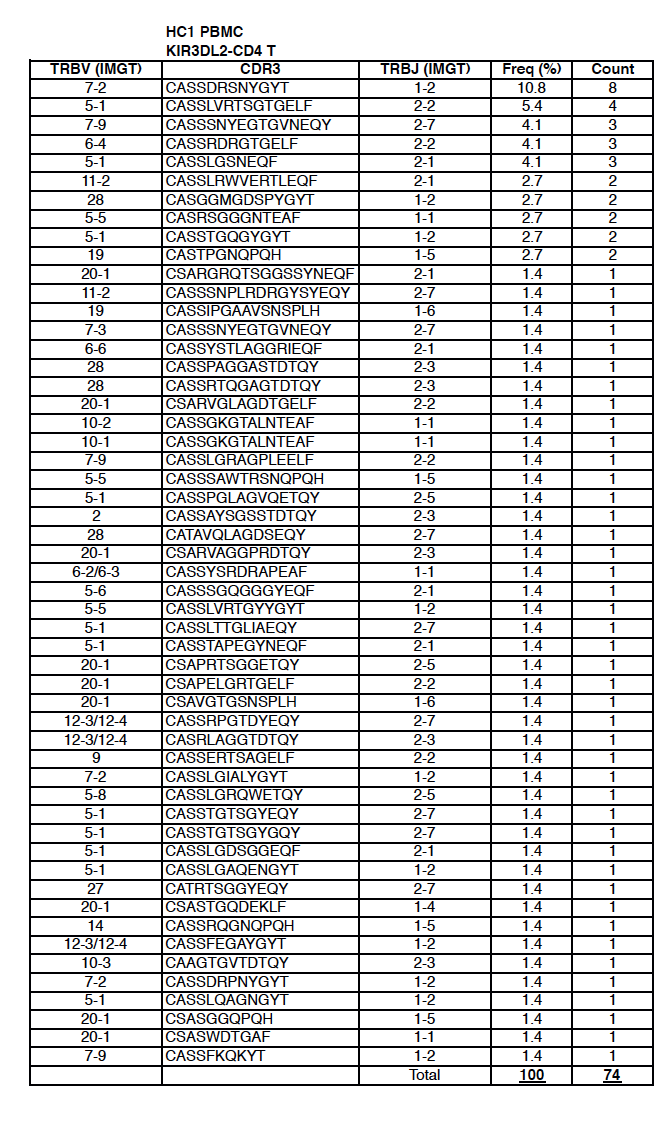
**

**
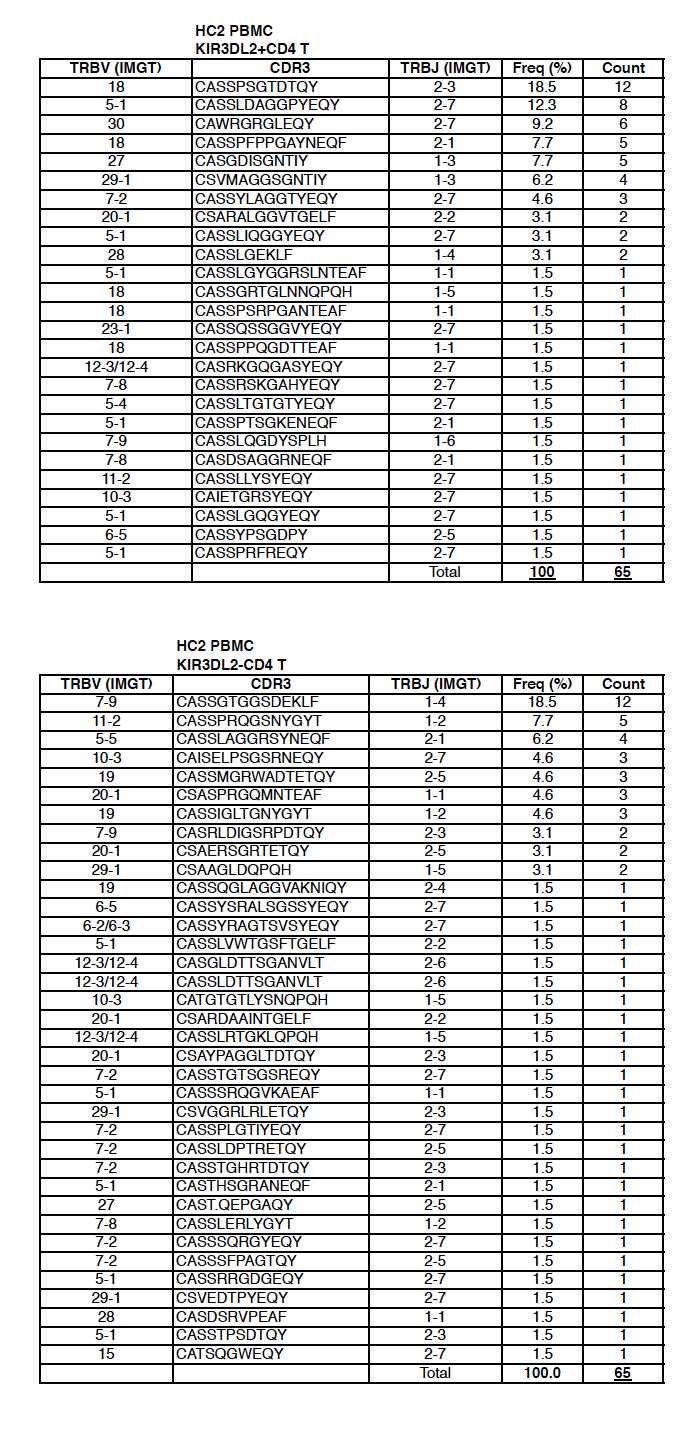
**

**
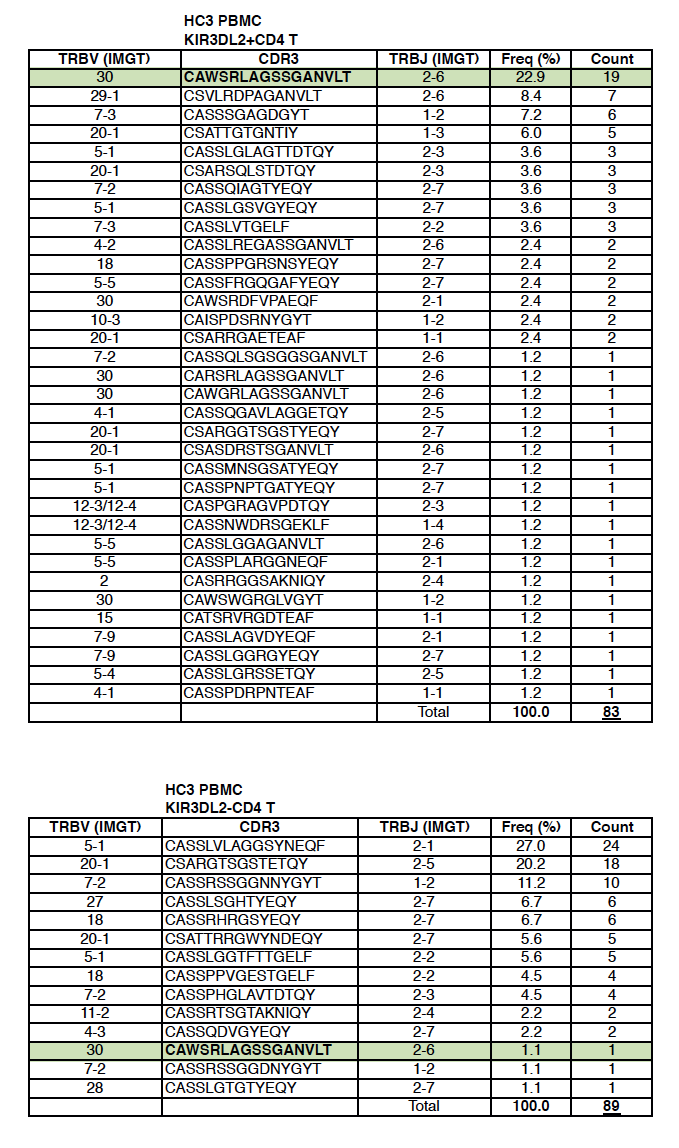
**
